# Quasi-Podands with 1,2,3-Triazole Rings from Bile Acid Derivatives: Synthesis, and Spectroscopic and Theoretical Studies

**DOI:** 10.1021/acs.joc.4c00195

**Published:** 2024-05-14

**Authors:** Anna Kawka, Hanna Koenig, Damian Nowak, Tomasz Pospieszny

**Affiliations:** † Department of Bioactive Products, Faculty of Chemistry, Adam Mickiewicz University, Uniwersytetu Poznańskiego 8 Street, 61-614 Poznań, Poland; ‡ Department of Quantum Chemistry, Faculty of Chemistry, Adam Mickiewicz University, Uniwersytetu Poznańskiego 8 Street, 61-614 Poznań, Poland

## Abstract

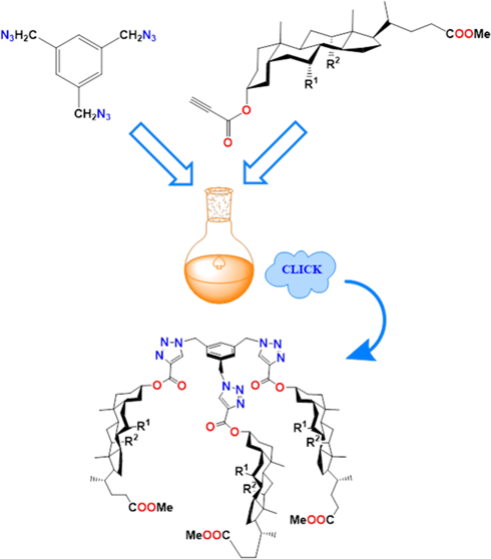

An innovative approach to producing derivatives of bile acids has been devised, utilizing the principles of “click” chemistry. By employing intermolecular [3 + 2] cycloaddition between the newly developed acyl propiolic esters of bile acids and the azide groups of 1,3,5-tris(azidomethyl)benzene, a novel class of quasi-podands featuring 1,2,3-triazole rings has been synthesized. Identifying and characterizing these six compounds involved comprehensive analysis through spectral techniques (^1^H NMR, ^13^C NMR, and FT-IR), mass spectrometry, and the PM5 semiempirical method. The synthesized compounds’ pharmacotherapeutic potential has been evaluated, employing the Prediction of Activity Spectra for Substances (PASS) methodology. Additionally, molecular docking was performed for all molecules.

## 1 Introduction

“Click” chemistry is one of the most modern paths of design and synthesis of new compounds that can be used in medicine, pharmacology, biotechnology, or supramolecular chemistry. Sharpless optimized this innovative method’s conditions for synthesizing new compounds used primarily as drugs.^1,2^

Obtaining new conjugates by “click” chemistry covers a broad spectrum of reactions leading to forming a carbon-heteroatom bond. Most importantly, it is characterized by extraordinary efficiency, selectivity, simple reaction conditions, and easy product isolation. Moreover, the product is stable in many solvents (e.g., in water).^3−5^

**Scheme 1:**
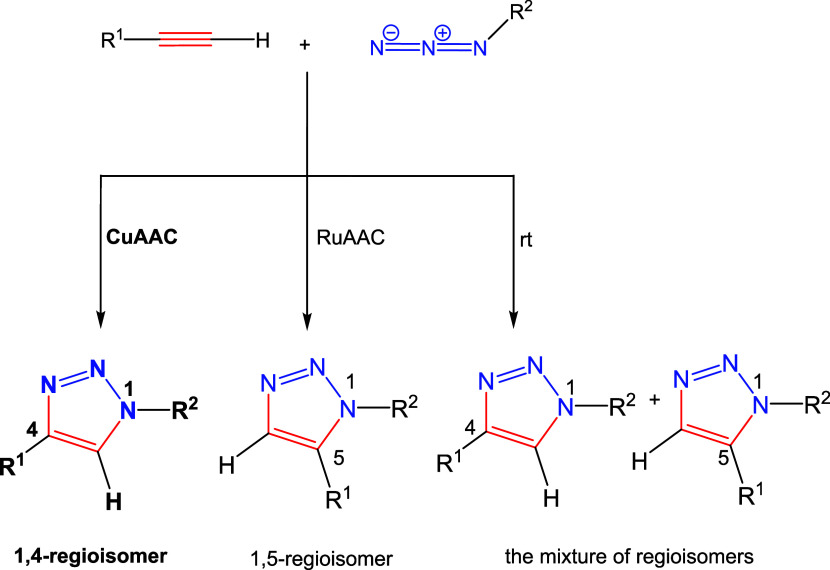
Visual Representation Showing the Different Possible Reaction Routes During a “Click” Reaction

[3 + 2] Cycloaddition catalyzed by copper(I) is a representative example of a reaction based on “click” chemistry conditions (Scheme 1). The Huisgen reaction between azides and terminal alkynes is considered the primary method for synthesizing 1,2,3-triazoles, characterized by antimicrobial and antitumor properties. In biological systems, these compounds show high resistance to hydrolysis, oxidation, reduction, or metabolic degradation reactions. In addition, they can interact with biological molecules by forming hydrogen bonds or dipole–dipole interactions. Moreover, the bonds that are formed are resistant to cleavage even by proteases. The result may be triazole systems peptide bond analogues.^6,7^ Literature data show that these are 1,2,3-triazole compounds characterized by unique pharmacotherapeutic effects, especially antihypertensive, antimalarial, antioxidant, antidepressant, antimicrobial, anti-inflammatory, and anticancer effects.^8−15^ The “click” method was used to obtain new steroid conjugates containing 1,2,3-triazole rings.

Compounds of natural origin affect the proper functioning of all cells in living organisms. Bile acids as representatives of steroids are responsible for the digestion and absorption of lipids in the small intestine, the right amount of cholesterol, glucose metabolism, or the composition of the intestinal microbiota. Their biosynthesis begins in liver cells by the oxidation of cholesterol by cytochrome P450, and the final products are stored in the form of conjugated bile salts with the amino acids glycine and taurine. Bile acids (lithocholic acid, deoxycholic acid, and cholic acid) are distinguished by a large, curved steroid skeleton, A/B rings in *cis* geometry, enantiomeric purity, a long chain attached to the C(17) atom with a carboxyl group, amphipathic and polar hydroxyl groups with different chemical reactivity (3α–OH > 7α–OH > 12α–OH).^16−18^ Their derivatives are valuable and essential for synthesizing macrocyclic compounds, steroid dimers, cholophanes, and drug-transporting molecules. Also, they can be used in designing molecular receptors capable of guest recognition in guest–host chemistry. Steroid conjugates have been used in medicine, pharmacology, supramolecular chemistry, biotechnology, and biomimetics.^19−22^

The growing interest in macromolecular derivatives of bile acids led to the development of the synthesis and use of molecular pockets^23−25^ and umbrellas.^26−28^ These compounds comprise at least two amphiphilic parts connected by a labile chain to the central atom. On the other hand, quasi-podands have a rigid benzene “platform” that allows them to obtain a conformer with appropriate geometry. Also, it can be crucial during interaction with the surface of biopolymers or acting as an “anchor” in biological systems.^29,30^ Such systems have found application as carriers of biological molecules, hydrogelators, organogelators, or artificial receptors.^31−37^

The combination of beneficial physicochemical properties of bile acids, triazole systems, and the aromatic ring may lead to the preparation of new quasi-podands with great application potential. It is worth noting that podands are simple analogues of crown ethers and cryptands; thus, they can be valuable ligands in forming stable complexes with monovalent cations.^38,39^ In addition, synthetic podands are easy to synthesize (as opposed to biological ones), and the spectrum of modification of their structures is endless.

## 2 Results and Discussion

### 2.1 Synthesis

**Scheme 2:**
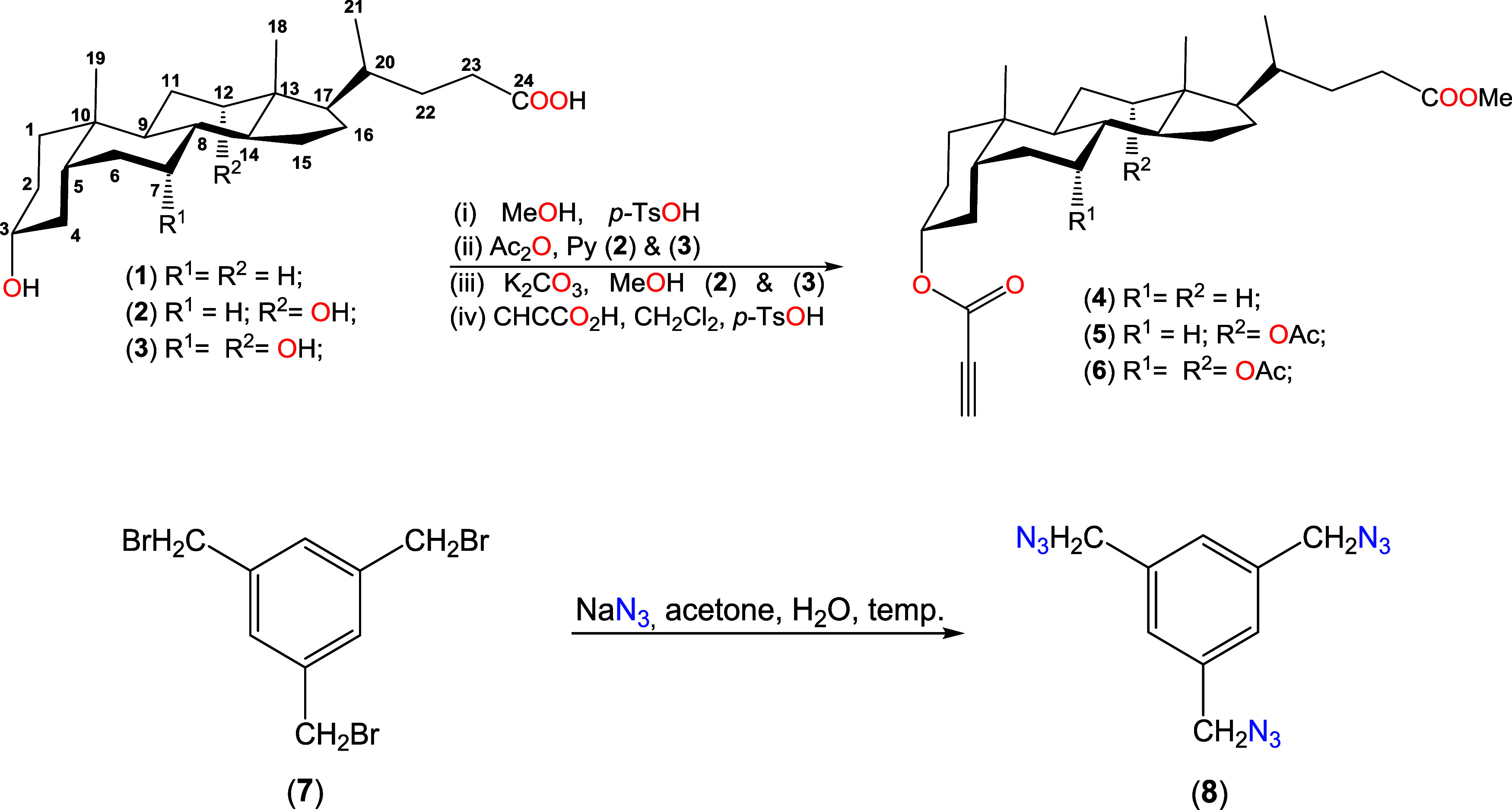
Synthesis of Propionyl Esters of Bile Acids (**4**–**6**) and 1,3,5-Tris(azidomethyl)benzene (**8**)

This study presents the synthesis and characterization of novel quasi-podands that are connected with a 1,2,3-triazole ring derived from propiolic esters of bile acids and 1,3,5-tris(azidomethyl)benzene. The propionyl esters of bile acids and 1,3,5-tris(azidomethyl)benzene were synthesized using previously established methods as described in Scheme 2.

**Scheme 3:**
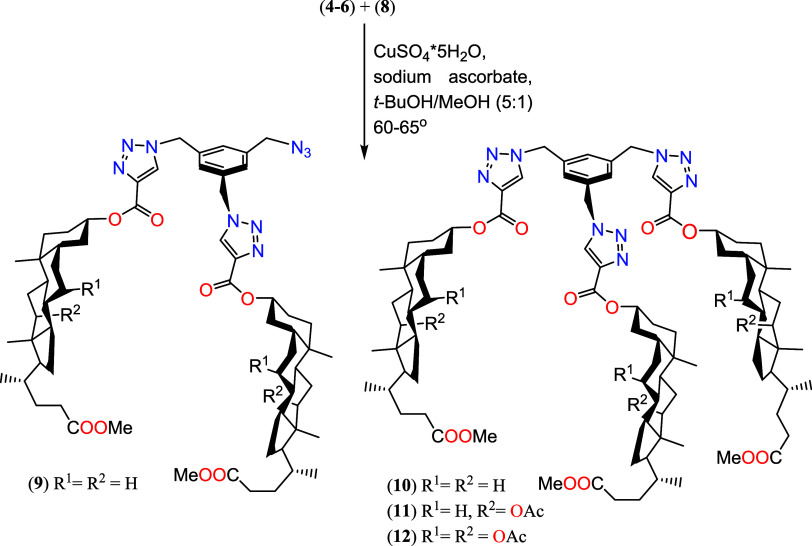
“Click” Synthesis of Quasi-Podands of Bile Acids Derivatives Linked by 1,2,3-Triazole Ring (**9**–**12**)

The synthetic procedures for compounds **9**–**12** are presented in Scheme 3. Our experiments yielded trisubstituted products **10**–**12** as well as one disubstituted product **9**, which were isolated and characterized. Unlike molecular pocket or umbrella structures, these conjugates possess a rigid benzyl platform. This structural feature facilitates the formation of conformers with favorable geometries. The flat aromatic ring enables effective interactions with various surfaces, including biopolymers, making it function as a specific anchor. Previous research by Ghosh et al. has described similar connections achieved using cholesterol derivatives. Interestingly, these compounds exhibit distinct gelling properties and detected ions Cu^2+^, Ag^+^, and Hg^2+^.^40^

### 2.2 Spectroscopic Characteristic

The structural characterization of all synthesized compounds was accomplished through analysis of their ^1^H and ^13^C NMR, FT-IR, and ESI-MS spectra. In addition, PM5 calculations were conducted for each compound to further explore their properties and characteristics.^41−43^

**Figure 1:**
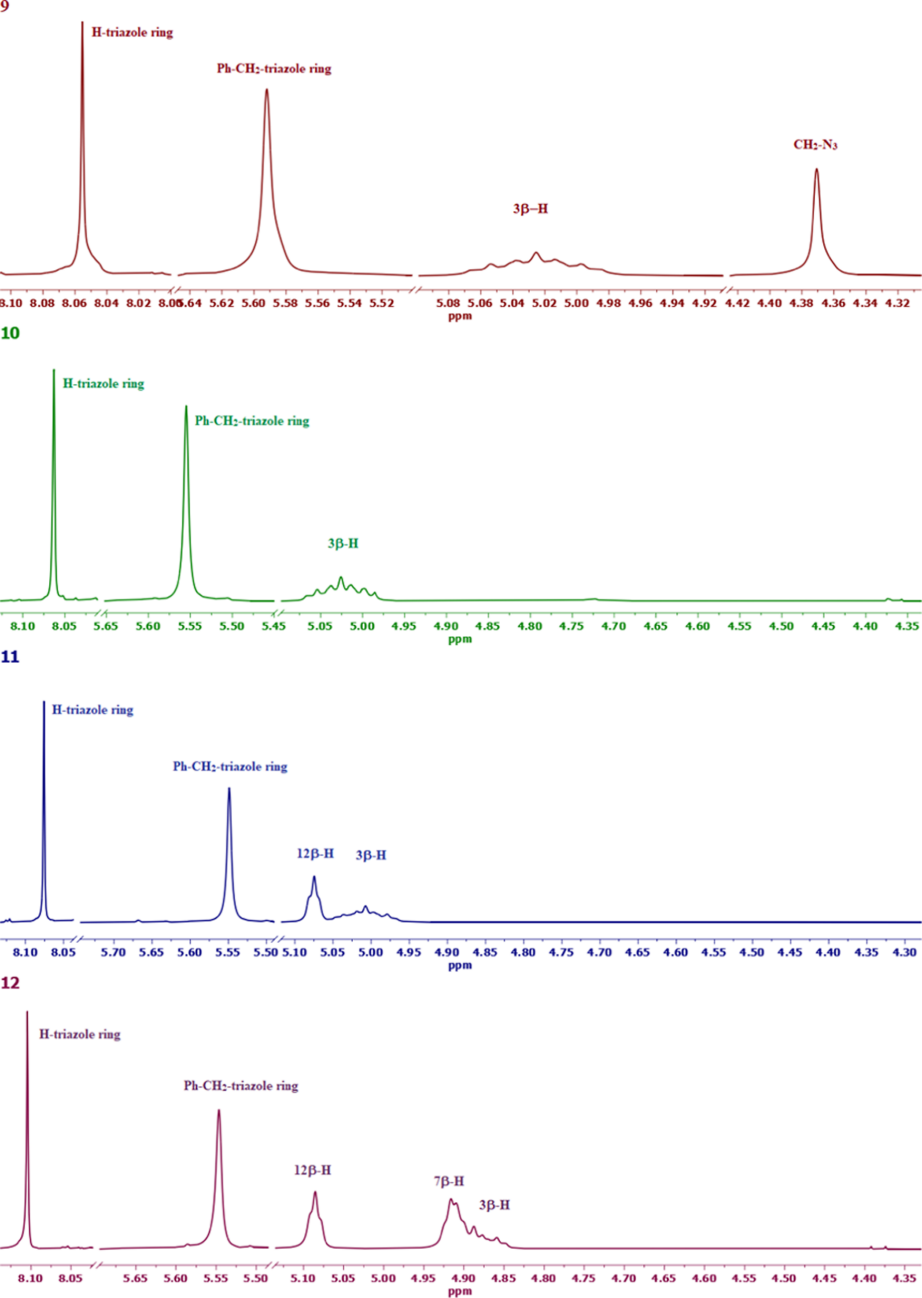
^1^H NMR spectra in the region (8.10–4.35 ppm) of the most characteristic signals of compounds (**9**–**12**).

The ^1^H NMR spectra of compounds **9** and **10**–**12** exhibit distinctive patterns of signals. Notably, in the range of 5.07–4.81 ppm, characteristic multiplets are observed, which can be attributed to the C3β-H protons of the steroid skeleton. Additionally, two hydrogen singlets appear in the ranges of 0.73–0.64 and 0.95–0.94 ppm, accompanied by characteristic doublets at 0.92–0.81 ppm, assigned to CH_3_-18, CH_3_-19, and CH_3_-21, respectively. In the spectra of compounds **11** and **12**, there are characteristic broad singlets observed in the range of 5.09–5.07 ppm, corresponding to the C12β-H protons. Furthermore, singlet in the range of 4.94–4.81 ppm is observed for the C7β-H protons in compound **12**. A distinctive signal at 4.37 ppm is observed in the ^1^H NMR spectra of compound **9**, which can be attributed to the protons of the −CH_2_–N_3_ group. This signal serves as a diagnostic marker and is absent in the spectra of compounds **10**–**12**. The singlet at 5.55 ppm represents the signal for the two methylene protons of the Ph–CH_2_–triazole ring group (refer to Figure 1).

Additionally, in the ^1^H NMR spectrum of compound **9**, there is a distinctive and diagnostically significant singlet observed at 8.06 ppm, which can be attributed to the two protons of the triazole rings. Conversely, in the spectra of compounds **11**–**12**, three protons of the triazole rings are observed in the range of 8.10–8.06 ppm. Moreover, the ^1^H NMR spectra of compounds **9**–**12** exhibit characteristic signals for the aromatic protons of the 1,3,5-trisubstituted benzene. These signals appear as singlets at 7.22–7.19 ppm for compound **9** and at 7.21–7.19 ppm for compounds **10**–**12**. These signals serve as prominent markers in the spectra of these compounds.

The ^13^C NMR spectra of compounds **9**–**12** exhibit distinct peaks at the following chemical shifts: 12.4–12.0, 23.4–22.8, and 18.2–17.4 ppm, which correspond to CH_3_-18, CH_3_-19, and CH_3_-21, respectively. However, the carbon atoms located in the 3α positions of the formyloxy groups exhibit resonance at 160.1–160.0 ppm. The carbon atoms within the C(12)=O steroid skeleton generate signals at 170.7 ppm, whereas the carbon atoms of C(7)=O are detected at 170.8 ppm. Alternatively, the carbon atoms of the C(24)=O group produce signals in the range of 174.8–174.5 ppm. The diagnostic signal for carbon atoms in the 1,2,3-triazole rings of compounds **9**–**12** is observed between 143.3–141.4 and 128.0–127.5 ppm, respectively. The carbon atoms within the CO_2_–CH_2_–triazole ring unit resonate within the range of 53.8–53.4 ppm (CH_2_). In the ^13^C NMR spectrum of compound **9**, the signal arising from the CH_2_ group in the N_3_–CH_2_–Ph moiety is observed at 53.7 ppm. The spectra of compounds **10**–**12** display signals associated with the CH_2_ atoms in the triazole ring-CH_2_–Ph structure.

The FT-IR spectra of compounds **5** and **6** exhibit notable features. These include bands at 3251, 3292, and 3286 cm^–1^, which are attributed to the stretching vibrations of the ν(≡C–H) group. The stretching vibrations of C–H bonds, forming a conjugate structure, merge into a broad band ranging from 2952 to 2869 cm^–1^. Another significant observation is the presence of important analytical bands at 1737–1734 cm^–1^, indicating the symmetric carbonyl group’s ν(C=O) stretching vibration in the FT-IR spectrum. Additionally, strong characteristic bands in the region 1247–1246 cm^–1^ can be observed, which are assigned to the ν(C–O) vibration.

**Figure 2:**
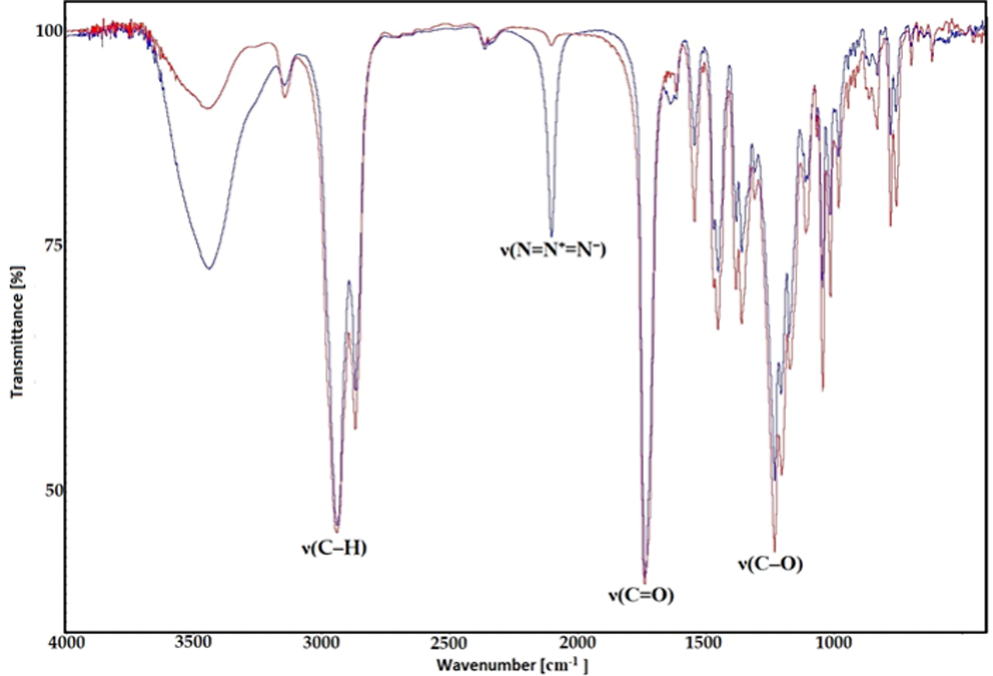
FT-IR spectra of **9** (blue) and **10** (red) in the region (3700–400 cm^–1^).

The FT-IR spectra of all synthesized compounds **9**–**12** exhibit a prominent feature characterized by bands at 2951–2866 cm^–1^, which are assigned to the stretching vibrations of the ν(C–H) groups. Additionally, two strong characteristic bands appear in the regions of 1736–1733 and 1246–1228 cm^–1^, which are attributed to the stretching vibrations of ν(C=O) and ν(C–O), respectively. Moreover, in the case of compound **9** a very strong band at 2099 cm^–1^ is observed, indicating the presence of ν(N = N^+^ = N^–^) groups (Figure 2).

**Figure 3:**
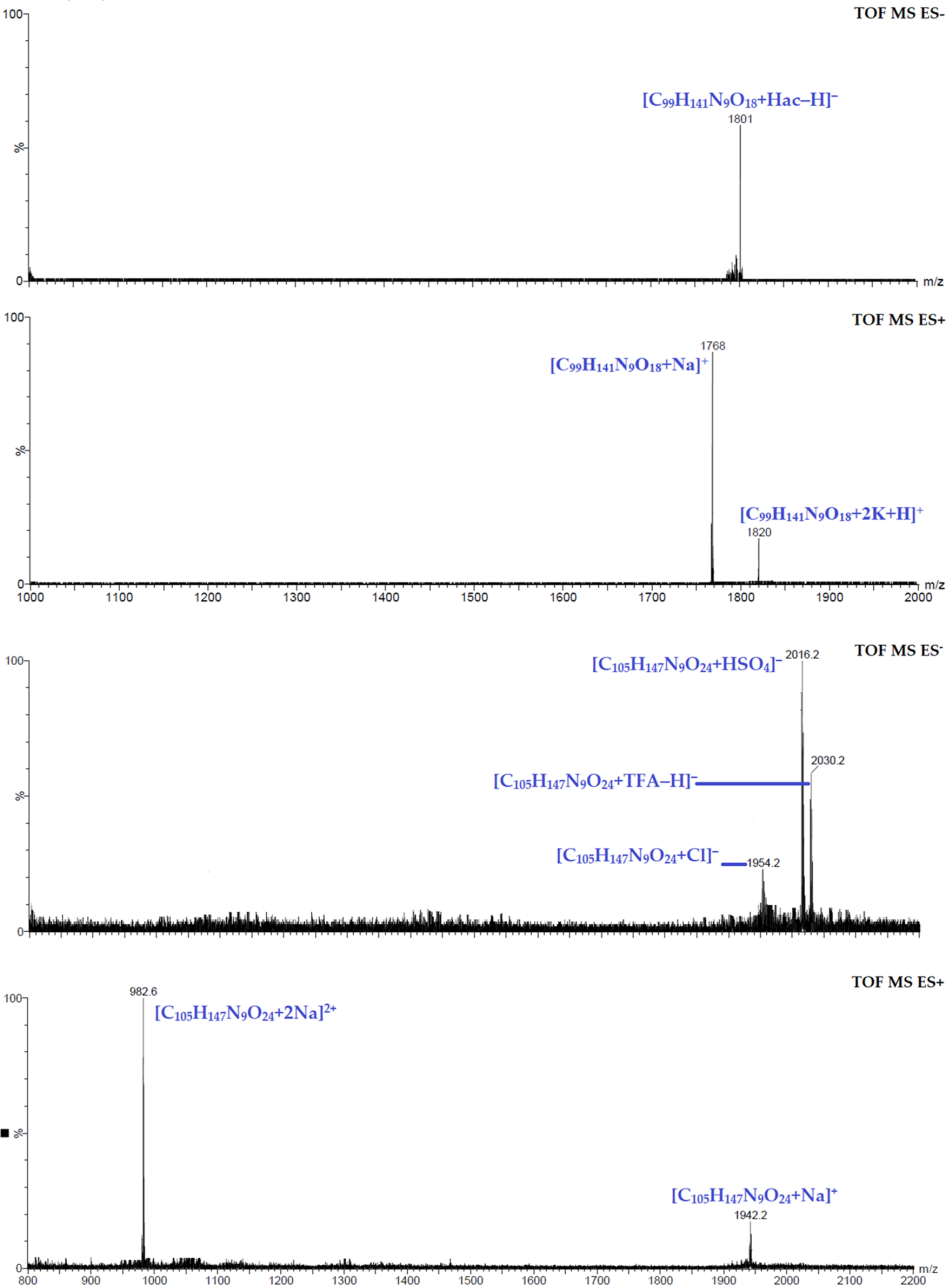
ESI-MS spectrum of conjugates **11** and **12**.

The ESI-MS spectra were acquired using methanol as the solvent. In all instances, the molecular ion [M]^+^ is detected, indicating the presence of a positively charged ion with a proton, alkali metals, or halides in positive-ion mode (ES^+^) as well as negative-ion mode (ES^–^). Figure 3 displays the ESI-MS spectrum of conjugates **11** and **12**. In this spectrum, ion peaks are observed at *m*/*z* 1820 (20%) [C_99_H_141_N_9_O_18_+2K+H]^+^, *m*/*z* 1768 (95%) [C_99_H_141_N_9_O_18_+Na]^+^ (for compound **11**), *m*/*z* 1942.2 (20%) [C_105_H_147_N_9_O_24_+Na]^+^, and *m*/*z* 982.6 (100%) [C_105_H_147_N_9_O_24_+2Na]^2+^ (for compound **12**). Furthermore, for these compounds, the ESI-MS spectrum in negative-ion mode exhibits the molecular ion at *m*/*z* 1801 (55%) [C_99_H_141_N_9_O_18_+Hac–H]^−^ (for **11**), *m*/*z* 2016.2 (100%) [C_105_H_147_N_9_O_24_+HSO_4_]^−^, *m*/*z* 1954.2 (25%) [C_105_H_147_N_9_O_24_+Cl]^−^, and *m*/*z* 2030.2 (55%) [C_105_H_147_N_9_O_24_+TFA–H]^−^ (for **12**) (Figure 3).

### 2.3 PM5 Calculations

The PM5 semiempirical calculations were performed using the WinMopac 2003 program. The final heat of formation (HOF) of compounds **5** and **6** as well as quasi-podands **9**–**13** are presented in Table 1.

**Table 1:** Heat of Formation (HOF) [kcal/mol] of Compounds**5**, **6**, and **9**–**13**

compound	heat of formation[kcal/mol]
**4**	–212.7209
**5**	–298.6465
**6**	–383.8356
**9**	–286.8410
**10**	–550.9153
**11**	–809.8199
**12**	–1063.6189

**Figure 4:**
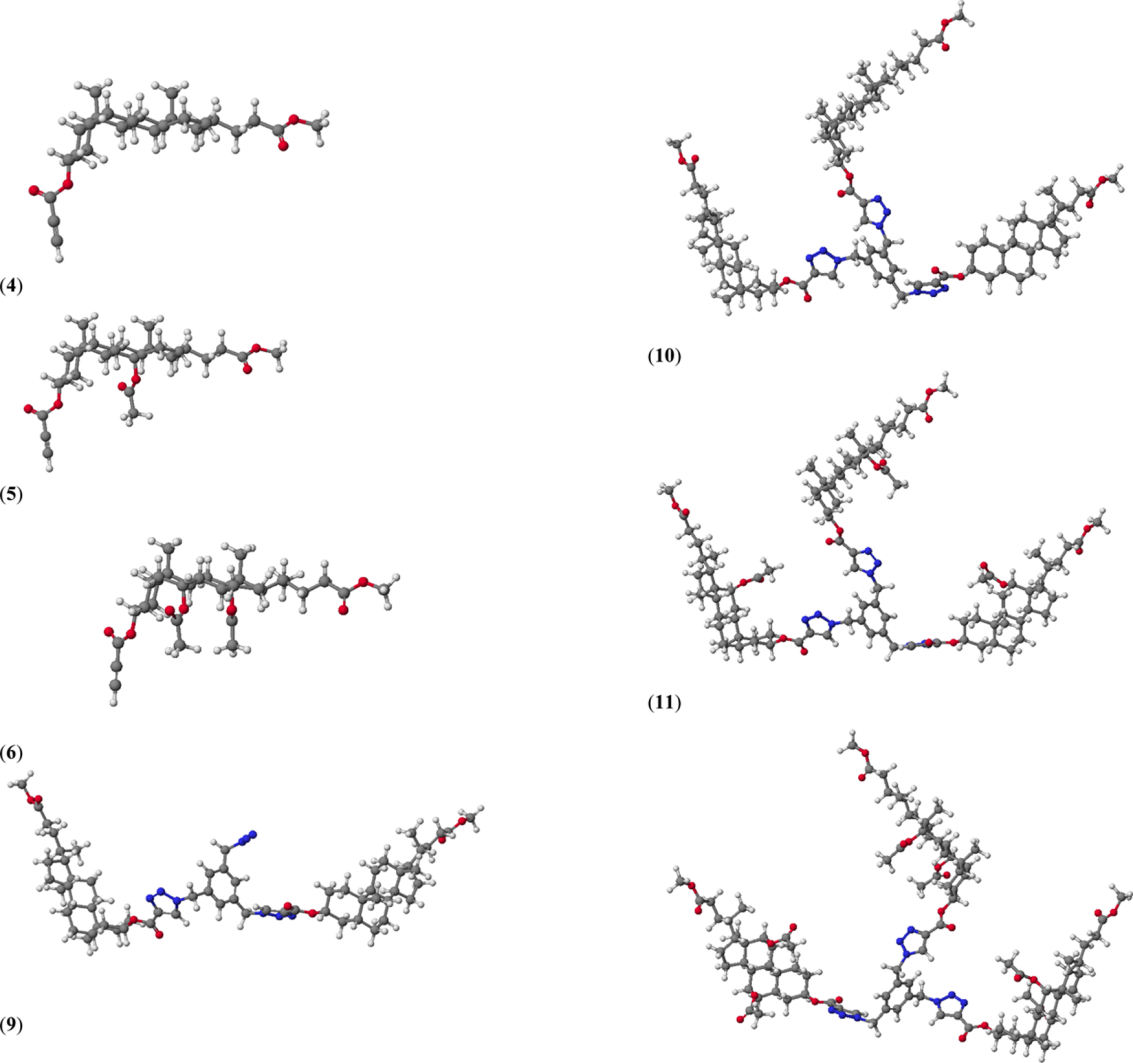
Molecular models of representative compounds **5** and **6** as well as **9**–**12** calculated by the PM5 method.

The molecular models of compounds **4**–**6** as well as **9**–**12** are shown in Figure 4. For the substrates **4**–**6**, the lowest values of HOF are observed for cholic acid derivatives **6**, where an increasing number of acetoxy groups facilitate the formation of intramolecular hydrogen bonds. It is noteworthy that monosubstituted derivatives of bile acids-linked 1,2,3-triazole ring (with two N_3_ groups) are not formed because the heat of formation is very high. Also, disubstituted derivatives are occasionally observed. Only lithocholic acid derivatives could be obtained, and their stability was still low (higher HOF than substrate **5**).

In all quasi-podands, we observed π–π stacking sandwich-type interactions between two triazole rings were observed. The calculated interplanar separation is about 5.8 Å. These distances are greater by about 1.7 Å in comparison to the classical π–π stacking interactions because the triazole ring is attached to directly a rigid aromatic ring which imposes an increasing distance. Furthermore, this spatial arrangement of bile acids and 1,2,3-triazole rings can facilitate the formation of stable host–guest complexes.

### 2.4 *In Silico* Biological Activity Studies

The pharmacological activity of the synthesized compounds **5**, **6**, and **9**–**12** has been assessed using computer-aided drug discovery methods, specifically employing the Prediction of Activity Spectra for Substances (PASSs) program. This program utilizes a comprehensive analysis of structure–activity relationships within a diverse training set containing approximately 60,000 biologically active compounds from various chemical series, encompassing around 4500 types of biological activity. By simply providing the structural formula of a chemical compound, the PASS prediction can be obtained, making it a valuable tool for initial investigations. Numerous instances exist where the implementation of the PASS approach has resulted in the identification of novel pharmacological agents.^44−47^

Furthermore, the analysis of the biological activity spectra for the newly two synthesized esters presented in this study serves as a notable illustration of *in silico* investigations on chemical compounds. The PASS program was employed to predict the biological activity spectra for two substrates **5** and **6** and one specific compound, namely, compound **9**. Focusing on the potential compound with the highest probability (referred to as focal activities) (see Table 2), we identified several frequently predicted types of biological activity, including acylcarnitine hydrolase inhibitor, alkenylglycerophosphocholine hydrolase inhibitor, alkylacetylglycerophosphatase inhibitor, dextranase inhibitor, and CYP2C and CYP2B6 substrates for **5** and **6**. On the other hand, for the quasi-podands, an activity of more than 60% but less than 70% was observed. The following can be mentioned here: glyceryl-ether monooxygenase inhibitor, antifertility (female), antienzematic, cholesterol antagonist, as well as cytoprotectant. However, due to their molecular weight exceeding 1200 g/mol, the potential biological properties of compounds **10**–**12** could not be determined in this analysis.

**Table 2:** PA (Probability “to be Active”) Values for the Predicted Biological Activity of Substrates**5** and **6** and Compounds**9**

focal predicted activity (PA > 80%) for **5** and **6**	compound		
focal predicted activity (PA > 60%) for **9**	**5**	**6**	**9**
glyceryl-ether monooxygenase inhibitor			66
antiinfertility (female)			62
antienzematic			67
cholesterol antagonist			60
cytoprotectant			60
acylcarnitine hydrolase inhibitor	96	96	
alkenylglycerophosphocholine hydrolase inhibitor	93	90	
alkylacetylglycerophosphatase inhibitor	93	90	
dextranase inhibitor	89	83	
CYP2C substrate	87	83	
CYP2B6 substrate	85	83	
respiratory analeptic	85		
analeptic	84		
protein-disulfide reductase (glutathione) inhibitor	84		
glyceryl-ether monooxygenase inhibitor	82	85	
peptidoglycan glycosyltransferase inhibitor	81		
CYP3A4 substrate	82	83	
CYP2C substrate		83	
flavin-containing monooxygenase inhibitor		82	
dextranase inhibitor		83	
hypolipemic		81	
adenomatous polyposis treatment		80	
CYP3A substrate		81	

### 2.5 Molecular Docking Studies

**Figure 5:**
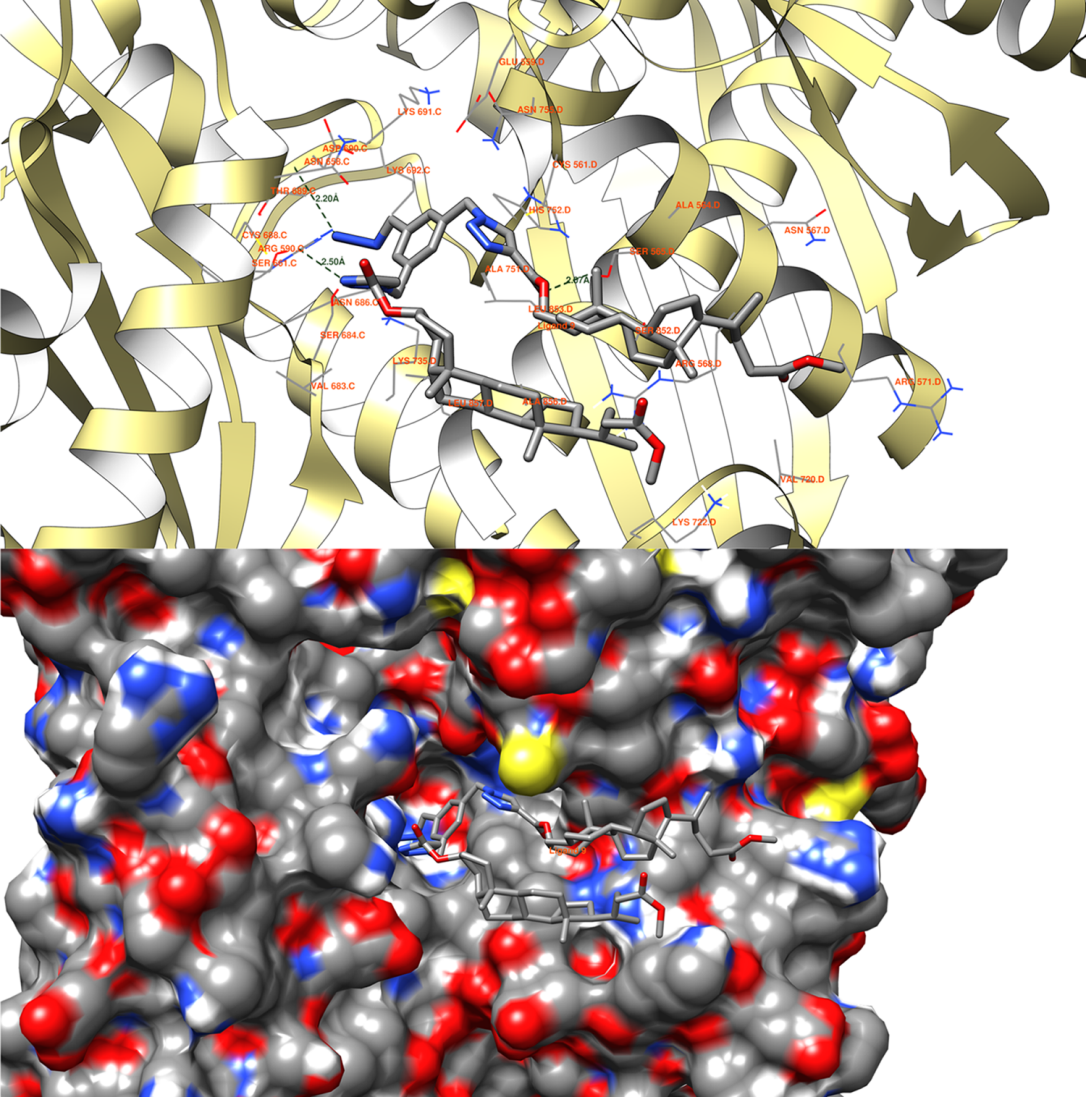
Ligand **9** possible hydrogen bonds between 1HW8 protein domain binding site. The binding energy equals −8.7 kcal/mol, with the average binding energy of −8.4 kcal/mol.

**Figure 6:**
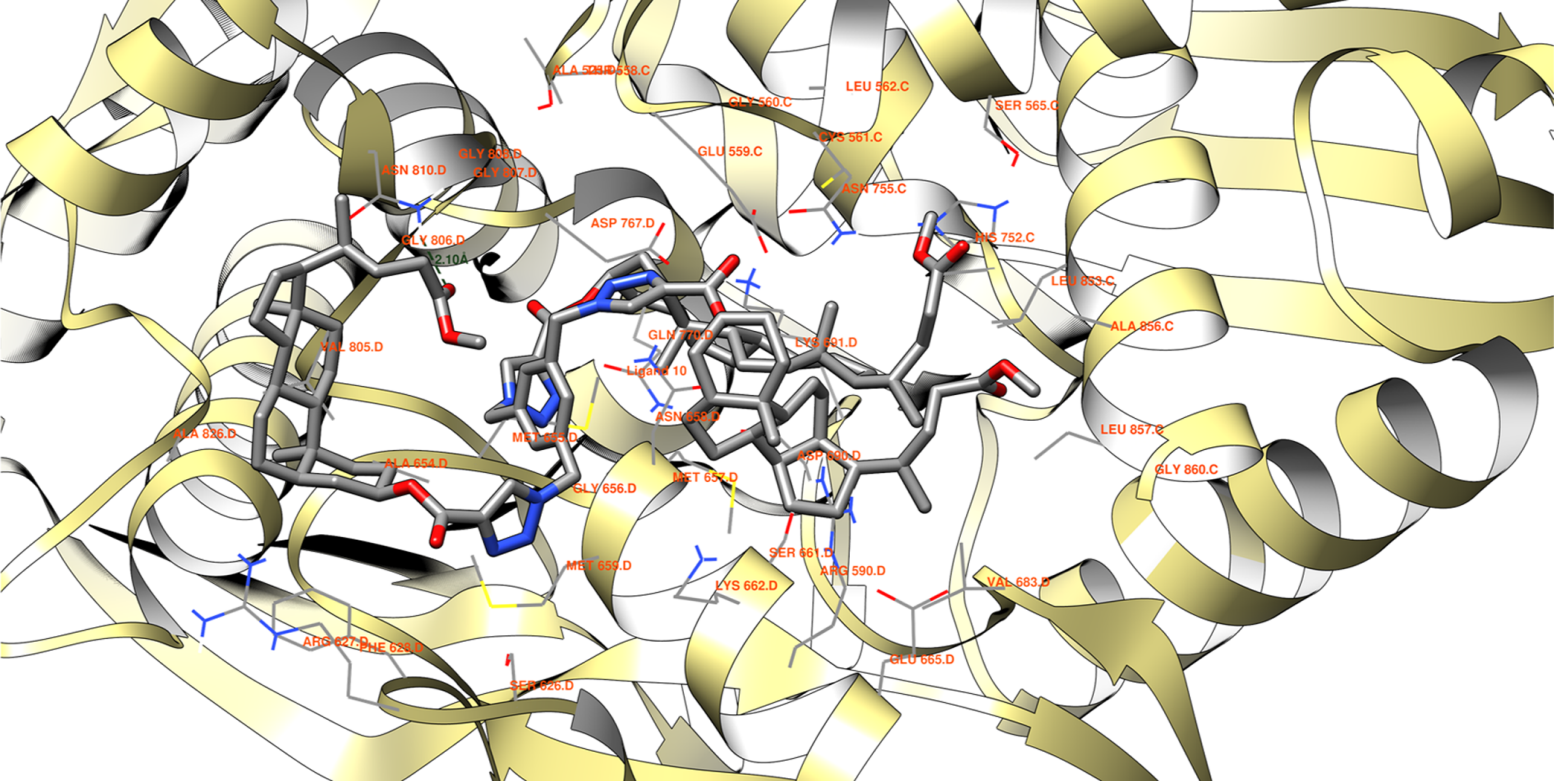
Ligand **10** possible hydrogen bonds between 1HW8 protein domain binding site. The binding energy equals −9.8 kcal/mol, with the average binding energy of −9.8 kcal/mol.

**Figure 7:**
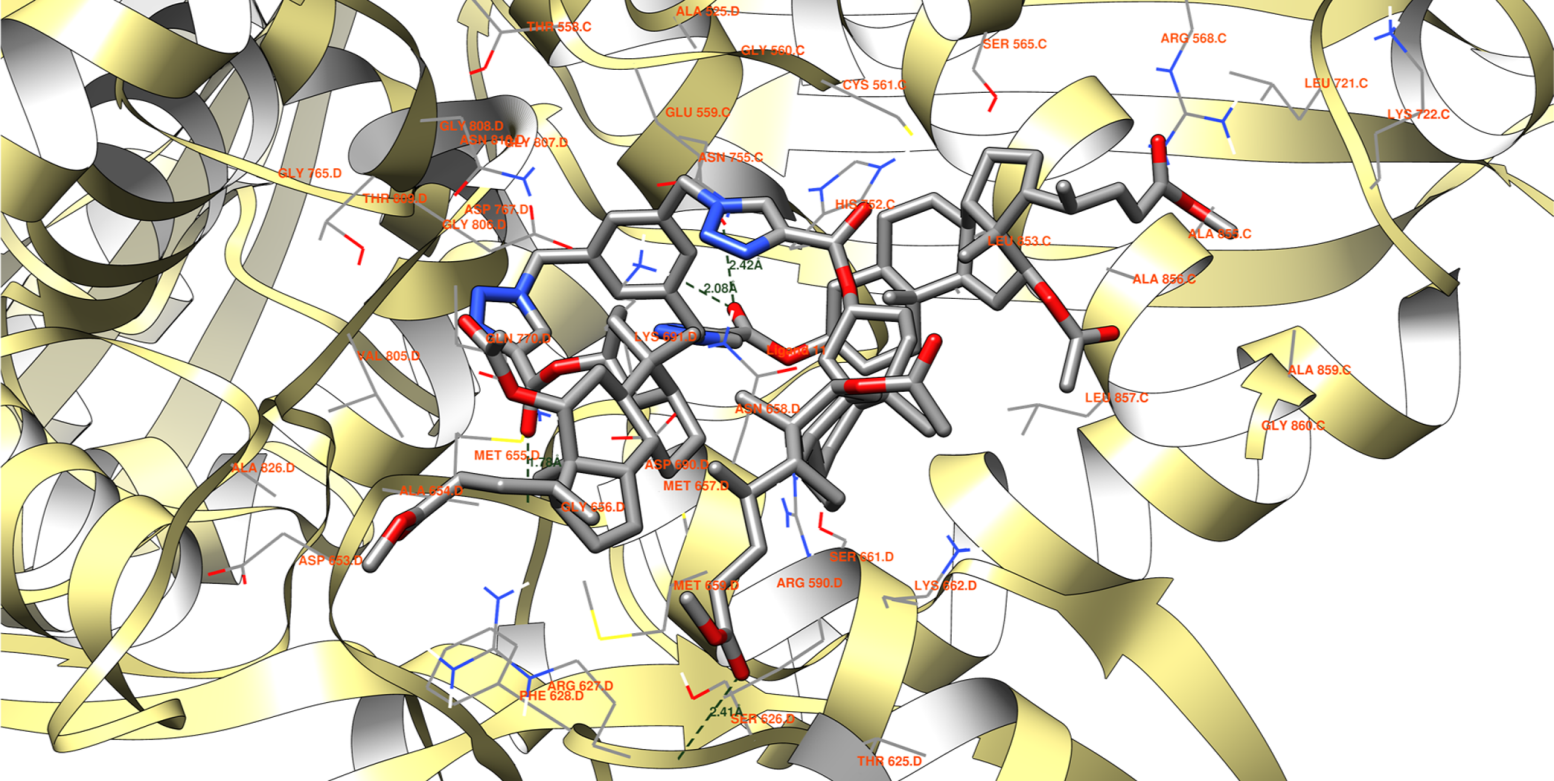
Ligand **11** possible hydrogen bonds between 1HW8 protein domain binding site. The binding energy equals −8.9 kcal/mol, with the average binding energy of −9.0 kcal/mol.

**Figure 8:**
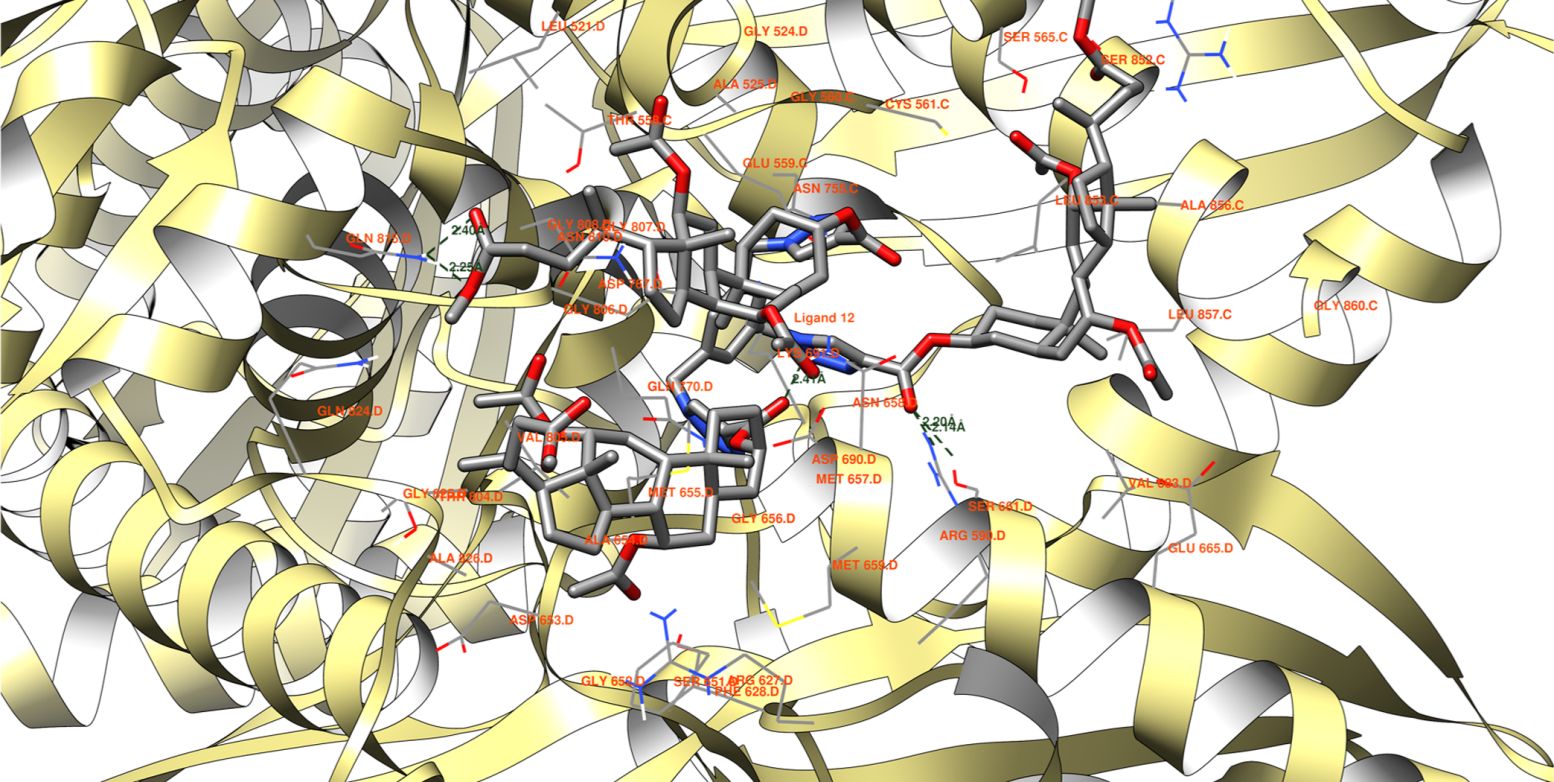
Ligand **12** possible hydrogen bonds between 1HW8 protein domain binding site. The binding energy equals −9.2 kcal/mol, with the average binding energy of −9.1 kcal/mol.

The macromolecular structure investigated in this study was identified by the PDB ID 1HW8. Below are the potential interactions observed between the analyzed structures and the protein domain. The graphical depictions illustrate the optimal ligand pose, determined by the binding site of the original ligands found within the raw PDB file 1HW8. These representations showcase the poses with the lowest binding energy observed inside the binding site. Figures 5–8 depict the interactions between the best poses of structures **9**, **10**, **11**, and **12**, respectively, and the protein domain of 1HW8.

For compound **9** (Figure 5), there exist three potential hydrogen bonds that could form between the ligand and the protein domain. The shortest among these potential interactions is identified between the ligand’s keto ester oxygen and the hydrogen of residue SER 565 D, spanning a length of 2.07 Å. The second possible hydrogen bond measures 2.20 Å and involves the azide group’s interaction with the hydrogen of residue ASP 690 C. The last interaction, furthest in distance, may occur between the ligand’s nitrogen within the 1,2,3-triazole group and the SER 661 C residue of the protein.

For compound **10** (Figure 6), the potential number of hydrogen bonds is two. The shorter interaction, measuring 2.10 Å, occurs between the ligand’s keto carboxyl oxygen and the hydrogen of residue ASN 810 D within the macromolecule.

According to the molecular docking studies, compound **11** (Figure 7) has the potential to form a total of four hydrogen bonds. These interactions are ranked in descending order of length:

- Another interaction involves the ligand’s ester oxygen forming a bond (2.42 Å) with the hydrogen of ASN 755 C, which competes with the hydrogen bond formation between the same ligand’s keto ester oxygen and the hydrogen of LYS 691 D residue (2.08 Å). The latter interaction is more favorable due to its shorter length.
- Additionally, another hydrogen bond is possible between another ligand’s keto ester oxygen and the hydrogen of ARG 627 D, measuring 2.41 Å.
- The shortest hydrogen bond for compound **11** spans a length of 1.78 Å and can be established between yet another ligand’s keto ester oxygen and the hydrogen of residue GLY 656 D.

The final compound investigated, ligand **12** (Figure 8), has the potential to form a total of five hydrogen bonds. However, four of these interactions compete with each other, resulting in the effective formation of three hydrogen bonds. The longest possible hydrogen bond exists between the ligand’s ester oxygen atom and the hydrogen of residue ASN 658 D.

Another competitive interaction involves the hydrogen of residue GLN 815 D forming a bond (2.40 Å) with the ketone oxygen of one of the ester groups. Additionally, an interaction occurs between the ether’s oxygen within the ligand’s ester group and the same hydrogen of the protein domain, measuring 2.25 Å. These interactions possess similar probabilities of formation due to their closely matching lengths.

The remaining two hydrogen bonds can be established between the ligand’s ester keto oxygen and the hydrogen of residue ARG 590 D (2.20 Å in length), as well as between the same oxygen atom of the ligand and a different residue, namely, SER 661 D. These interactions similarly exhibit very close probabilities of formation.

The docked molecules exhibit a higher affinity toward the 1HW8 protein domain compared to the cocrystallized ligand, which demonstrated a binding energy of −7.5 kcal/mol, with an average binding energy of −6.2 kcal/mol. Similarly, they display a higher affinity compared to mevastatin, showing a binding energy of −6.9 kcal/mol, with an average binding energy of −6.8 kcal/mol. This suggests that these molecules have the potential to serve as effective inhibitors. However, it is essential to note that the sizes of ligands **9** through **12** are notably larger than those of the known activity inhibitors used for comparison, potentially influencing the sensitivity of this comparison. Figures 5–8 display binding energies, denoted in kcal/mol units (specified in the figures’ descriptions).

## 3 Conclusions

In summary, an efficient synthesis of two new propiolic derivatives and four innovative bile acid bioconjugates with 1,2,3-triazole rings (compounds **9**–**12**) was designed using the “click” chemistry method. The reaction of propiolic or acyl propionic bile acid derivatives with 1,3,5-tris(azidomethyl)benzene in a mixture of *tert*-butanol/methanol with the addition of sodium ascorbate and CuSO_4_*5 H_2_O at 60–65 °C yielded macrocyclic compounds containing rings 1,2,3-triazoles. The newly synthesized compounds were thoroughly characterized using spectroscopic techniques and molecular structure analysis. Additionally, the performed molecular docking indicates the potential inhibitory properties of the obtained structures. The Nobel Prize in Chemistry awarded for the study of “click” chemistry serves as conclusive evidence of the significance of this approach in organic synthesis. The growing interest in utilizing “click” chemistry for the synthesis of novel bioconjugates involving bile acids and sterols has a profound impact on the advancement of supramolecular chemistry, pharmacology, and medicine. The design and preparation of compounds incorporating the 1,2,3-triazole ring offer immense biological potential and a diverse range of physicochemical properties, enabling their application as artificial receptors, organogels, and novel complexing and drug delivery agents.

## 4 Experimental Section

### 4.1 Synthesis

#### 4.1.1 General Procedure for the Synthesis of Compounds **4**–**6**

The methyl esters of bile acids were prepared according to a previously described procedure. In the case of compounds **4**, **5**, or **6**, the bile acids (1 equiv) were dissolved in 15 mL of dichloromethane. Subsequently, *p*-TsOH and propiolic acid (3 equiv) were added to the solution, and the reaction was allowed to proceed for 24 h at room temperature. After completion of the reaction, the mixture was subjected to a series of purification steps. It was first washed with cool water, followed by extraction with chloroform (20 mL). The chloroform layer was then washed with water and brine, and dried using Na_2_SO_4_. Finally, the solvent was removed under reduced pressure, yielding the following product yields: (45%) for **4**, (57%) for **5**, and (30%) for **6**.

#### 4.1.2 General Procedure for the Synthesis of Compounds **9**–**10**

1,3,5-Tris(azidomethyl)benzene (30 mg, 0.123 mmol) was dissolved in a mixture of *tert*-butanol and methanol (12 mL, 5:1). Propiolic lithocholic ester (163 mg, 0.369 mmol) was then added, and the resulting mixture was heated at 60–65 °C (in a water bath) for 30 min. Next, to the homogeneous solution, CuSO_4_*5H_2_O (3 mg, 3 mol %) and sodium ascorbate (9 mg, 20 mol %) in water (0.3 mL) were added. The mixture was further heated to 60–65 °C (in a water bath) for 4 h. The resulting mixture was extracted with chloroform (10 mL), washed with brine (15 mL), and dried using anhydrous Na_2_SO_4_. After evaporating the solvent and purifying the residue over silica gel (CHCl_3_/EtOAc, 25:1), 20.3 mg (11%) of product **9** and 169.7 mg (88%) of product **10** were obtained.

##### Methyl 3α-Propynoyloxy-12α-acetoxy-5β-cholan-24-oate **(5)**

Oil (130 mg, 57%). ^1^H NMR (400 MHz, CDCl_3_): δ ppm 5.08 (s, 1H, 12β-H), 4.88–4.80 (m, 1H, 3β-H), 3.66 (s, 3H, –OCH_3_), 2.89 (s, 1H, –C≡CH), 2.11 (s, 3H, 12α-OAc), 0.91 (s, 3H, CH_3_-19), 0.80 (d, *J* = 6.3 Hz, 3H, CH_3_-21), 0.72 (s, 3H, CH_3_-18). ^13^C {^1^H} NMR (101 MHz, CDCl_3_) δ 174.6 (C-24), 170.5 (CO-12α), 152.1 (C-26), 75.8 (C-12), 75.8 (≡CH), 75.0 (C-3), 74.2 (–C≡), 51.5 (C-25), 49.3, 47.5, 45.0, 41.8, 35.6, 34.6, 34.6, 34.4, 34.0, 31.9, 30.9, 30.8, 27.2, 26.8, 26.3, 25.8, 25.6, 23.4, 23.0 (C-19), 21.4 (OAc-12α), 17.5 (C-21), 12.4 (C-18). FT-IR (KBr, cm^–1^) ν_max_: 3445, 3251, 2938, 2869, 2112, 1734, 1701, 1377, 1246, 1194. ESI-MS *m*/*z*: 523 [M + Na]^+^, 539 [M + K]^+^.

##### Methyl 3α-Propynoyloxy-7α,12α-diacetoxy-5β-cholan-24-oate **(6)**

Oil (153 mg, 30%), ^1^H NMR (400 MHz, CDCl_3_): δ ppm 5.09 (s, 1H, 12β-H), 4.92–4.91 (d, *J =* 3.1 Hz, 1H, 7β-H), 4.76–4.68 (m, 1H, 3β-H), 3.66 (s, 3H, –OCH_3_), 2.91 (s, 1H, –C≡CH), 2.15 (s, 1H, 7α-OAc), 2.09 (s, 1H, 12α-OAc), 0.92 (s, 3H, CH_3_-19), 0.81 (d, *J =* 6.4 Hz, 3H, CH_3_-21), 0.73 (s, 3H, CH_3_-18). ^13^C {^1^H} NMR (101 MHz, CDCl_3_) δ 174.5 (C-24), 170.5 (CO-12α), 170.4 (CO-7α), 152.0 (C-26), 75.3 (C-12), 75.3 (≡CH), 75.0 (C-3), 74.4 (–C≡), 70.5 (C-7), 51.2 (C-25), 47.3, 45.0, 43.4, 40.9, 37.7, 34.6, 34.5, 34.3, 34.2, 31.2, 30.8, 30.7, 29.7, 28.9, 27.1, 26.5, 25.6, 22.8, 22.4 (C-19), 21.6 (OAc-7α), 21.5 (OAc-12α), 17.5 (C-21), 12.2 (C-18). FT-IR (KBr, cm^–1^) ν_max_: 3247, 2952, 2873, 2116, 1737, 1439, 1378, 1247. ESI-MS *m*/*z*: 581 [M + Na]^+^, 597 [M + K]^+^, 594 [M + Cl]^−^.

##### 1-Azidomethylene-3,5-di[2-(methyl 5β-cholan-24-oate)-2-oxoethyl-1H-1,2,3-triazole- 4-(3-carboxylate)]benzene **(9)**

Oil (34 mg, 11%). ^1^H NMR (400 MHz, CDCl_3_): δ ppm 8.06 (s, 2H, triazole ring), 7.22 and 7.19 (s, 3H, Ar–H), 5.59 (s, 4H, Ph–CH_2_–triazole ring), 5.07–4.99 (m, 2H, 3β-H), 4.37 (s, 2H, CH_2_–N_3_), 3.67 (s, 6H, –OCH_3_) 0.95 (s, 6H, CH_3_-19), 0.92 (d, *J =* 6.5 Hz, 6H, CH_3_-21), 0.64 (s, 6H, CH_3_-18). ^13^C {^1^H} NMR (101 MHz, CDCl_3_) δ 174.8 (C-24), 160.0 (C-26), 141.3 (C-27), 138.3 (Ar–CCH_2_–triazole ring), 136.0 (Ar–CCH_2_N_3_), 127.9 (C-28), 127.5 (C–Ar), 127.2 (C–Ar), 75.7 (C-3), 56.4, 56.0, 53.8 (C-29), 53.7 (C-29’), 51.5 (C-25), 42.7, 41.9, 40.4, 40.1, 35.8, 35.3, 35.0, 34.6, 32.1, 31.0, 28.2, 27.0, 26.5, 26.4, 26.3, 24.2, 23.2 (C-19), 20.8, 18.2 (C-21), 12.0 (C-18). FT-IR (KBr, cm^–1^) ν_max_: 2933, 2866, 2099, 1733, 1228, 1203, 1043. ESI-MS (*m*/*z*): 1225 [C_65_H_93_N_9_O_8_+HSO_4_]^−^, 393 [C_65_H_93_N_9_O_8_+H+2Na]^3+^, 413 [C_65_H_93_N_9_O_8_+3K]^3+^, 565 [C_65_H_93_N_9_O_8_+2H]^2+^.

##### 1,3,5-Tris[2-(methyl 5β-cholan-24-oate)-2-oxoethyl-1H-1,2,3-triazole-4-(3-carboxylate)] benzene **(10)**

Crystal (170 mg, 88%), mp 130–133 °C. ^1^H NMR (400 MHz, CDCl_3_): δ ppm 8.06 (s, 3H, triazole ring), 7.20 (s, 3H, Ar–H), 5.55 (s, 6H, Ph–CH_2_–triazole ring), 5.07–4.99 (m, 3H, 3β-H), 3.67 (s, 9H, OCH_3_), 0.95 (s, 9H, CH_3_-19), 0.92 (d, *J =* 6.5 Hz, 9H, CH_3_-21), 0.65 (s, 9H, CH_3_-18). ^13^C {^1^H} NMR (101 MHz, CDCl_3_) δ 174.7 (C-24), 160.0 (C-26), 141.4 (C-27), 136.6 (Ar–C), 127.8 (CH-Ar), 127.5 (C-28), 75.8 (C-3), 56.4, 56.0, 53.4 (C-29), 51.5 (C-25), 42.7, 41.9, 40.3, 40.1, 35.8, 35.3, 35.0, 34.6, 32.1, 31.0, 31.0, 28.1, 27.0, 26.5, 26.3, 24.2, 23.2 (C-19), 21.0, 18.2 (C-21), 14.2, 12.0 (C-18). FT-IR (KBr, cm^–1^) ν_max_: 2939, 2866, 1736, 1228, 1040. ESI-MS (*m*/*z*): 533 [C_93_H_135_N_9_O_12_+2H+Na]^3+^, 810 [C_93_H_135_N_9_O_12_+2Na]^2+^.

##### 1,3,5-Tris[2-(methyl 12α-acetoxy-5β-cholan-24-oate)-2-oxoethyl]-1H-1,2,3-triazole-4-(3-carboxylate)benzene **(11)**

Crystal (138 mg, 77%,), mp 136–139 °C. ^1^H NMR (400 MHz, CDCl_3_): δ ppm 8.08 (s, 3H, triazole ring), 7.19 (s, 3H, Ar–H), 5.55 (s, 6H, Ph–CH_2_–triazole ring), 5.07 (d, 3H, 12β-H), 5.05–4.96 (m, 3H, 3β-H), 3.67 (s, 9H, OCH_3_), 2.12 (s, 9H, 12α-OAc), 0.94 (s, 9H, CH_3_-19), 0.80 (d, *J =* 6.3 Hz, 9H, CH_3_-21), 0.73 (s, 9H, CH_3_-18). ^13^C {^1^H} NMR (101 MHz, CDCl_3_) δ 174.6 (C-24), 170.7 (C-31), 160.0 (C-26), 141.4 (C-27), 136.5 (Ar–C), 127.8 (Ar–CH), 127.7 (C-28), 75.8 (C-3 and C-12), 53.4 (C-29), 51.5 (C-25), 49.3, 47.5, 45.0, 42.0, 36.5, 34.2, 32.2, 30.9, 30.8, 27.3, 27.0, 26.6, 25.7, 23.4 (C-19), 23.1, 21.5 (OAc-12α), 17.5 (C-21), 12.4 (C-18). FT-IR (KBr, cm^–1^) ν_max_: 2950, 2869, 1736, 1672, 1245, 1195, 1041, 1020. ESI-MS (*m*/*z*): 1801 [C_99_H_141_N_9_O_18_+Hac–H]^−^, 1768 [C_99_H_141_N_9_O_18_+Na]^+^. 1820 [C_99_H_141_N_9_O_18_+2K+H]^+^.

##### 1,3,5-Tris[2-(methyl 7α,12α-diacetoxy-5β-cholan-24-oate)-2-oxoethyl]-1H-1,2,3-triazole-4-(3-carboxylate)benzene **(12)**

Crystal (176 mg, 57% yield), mp 136–139 °C. ^1^H NMR (400 MHz, CDCl_3_): δ ppm 8.10 (s, 3H, triazole ring), 7.21 (s, 3H, Ar–H), 5.55 (s, 6H, Ph–CH_2_–triazole ring), 5.09 (d, 3H, 12β-H), 4.94–4.81 (m, 6H, 3β-H i 7β-H), 3.67 (s, 9H, OCH_3_), 2.20 (s, 9H, 7α-OAc) 2.08 (s, 9H, 12α-OAc), 0.95 (s, 9H, CH_3_-19), 0.81 (d, *J =* 6.2 Hz, 9H, CH_3_-21), 0.73 (s, 9H, CH_3_-18). ^13^C {^1^H} NMR (101 MHz, CDCl_3_) δ 174.5 (C-24), 170.8 (C-33), 170.6 (C-31), 160.1 (C-26), 141.4 (C-27), 136.5 (C–Ar), 128.0 (C-28 i CH-Ar), 75.7 (C-12), 75.3 (C-3), 70.5 (C-7), 53.4 (C-29), 51.5 (C-25), 47.3, 45.1, 43.4, 41.0, 37.7, 34.6, 31.3, 31.8, 30.7, 29.0, 27.1, 26.7, 25.7, 22.8 (C-19), 22.5 (OAc-7α), 21.7 (OAc-12α), 17.4 (C-21), 12.2 (C-18). FT-IR (KBr, cm^–1^) ν_max_: 2951, 2872, 1735, 1378, 1246, 1022. ESI-MS (*m*/*z*): 2016.2 [C_105_H_147_N_9_O_24_+HSO_4_]^−^, 1954.2 [C_105_H_147_N_9_O_24_+Cl]^−^, 1942.2 [C_105_H_147_N_9_O_24_+Na]^+^, 982.6 [M+2Na]^2+^, 2030.2 [C_105_H_147_N_9_O_24_+TFA–H]^−^.

### 4.2 Molecular Docking Studies

The molecular docking process utilized the OpenBabel software^48,49^ to generate three-dimensional (3D) structures from SMILES representations of compounds. These structures were initially saved in *.pdb format and subsequently converted to the required *.pdbqt format for compatibility with the AutoDock Vina algorithm.^50^ The receptor, represented by 1HW8 (PDB ID, HMG-CoA reductase), was prepared using AutoDock Tools 1.5.7.^51,52^ The molecular docking methodology employed the AutoDock Vina algorithm utilizing the multiple CPU technique.^49^ Visualizations of the best poses of the docked ligands and potential hydrogen bond formations were performed using the Chimera tool (version 1.16).^53^

In this study, the receptor 1HW8 (PDB ID, HMG-CoA reductase) was selected for docking structures **9**, **10**, **11**, and **12** (refer to Figure 8), acquired from the Protein Data Bank (PDB).^54−56^ This enzyme is pivotal in cholesterol production within the liver. Inhibiting the HMG-CoA reductase activity can potentially reduce cholesterol production, subsequently lowering its concentration in the bloodstream.^56^

The docked structures were targeted to the active site of the protein domain, analogous to the cocrystallized ligands. The search parameters utilized for 1HW8 were set as follows: center (*x*, *y*, *z*): 22.145, 21.402, 29.764, and size (*x*, *y*, *z*) (80 × 80 × 80) Å^∧^3.

## Data Availability

The data underlying this study are available in the published article and its Supporting Information.

## Supporting Information Available

General information and copies of ESI-MS, ^1^H and ^13^C NMR, and FT-IR spectra (PDF)

## Notes

**Safety Statement**: The obtained compounds were obtained under laboratory conditions. The compounds have not been subjected to cytotoxicity tests; therefore, their effect on human or animal cells is not known.
