# Supplementary material for: Quasi-Podands with 1,2,3-Triazole Rings from Bile Acid Derivatives: Synthesis, and Spectroscopic and Theoretical Studies
Source: J Org Chem. 2024 May 14;89(11):7561–72. doi: 10.1021/acs.joc.4c00195 (PMC11165584; doi:10.1021/acs.joc.4c00195)
Supplement: Supplementary file 1 — jo4c00195_si_001.pdf [file jo4c00195_si_001.pdf]

Supporting Information

Quasi-Podands with 1,2,3-Triazole Rings from Bile Acid Derivatives: Synthesis, Spectroscopic and Theoretical Studies

Anna Kawka[a], Hanna Koenig[a], Damian Nowak[b], Tomasz Pospieszny[a*]

[a]*Department of Bioactive Products, Faculty of Chemistry, Adam Mickiewicz University,*

Uniwersytetu Poznańskiego 8 Street, 61-614 Poznań, Poland

[b]*Department of Quantum Chemistry, Faculty of Chemistry, Adam Mickiewicz University, Uniwersytetu Poznańskiego 8 Street, 61-614 Poznań, Poland*

*Corresponding Author: tposp@amu.edu.pl

Table of Contents

1. General Information S2
2. Copies of NMR spectra S3
3. Copies of ESI-MS spectra S9
4. Copies of FT-IR spectra S11

1. General Information

The following reagents were acquired from Sigma-Aldrich Corporation for the synthesis: lithocholic acid, deoxycholic acid, cholic acid, acetic anhydride, pyridine, propiolic acid, sodium azide, and sodium ascorbate. Chloroform, dichloromethane, *t*-butanol, and methanol solvents were obtained from standard commercial sources such as Merck and Fisher, and were used without purification. The characterization methods used are as follows:

IR Spectra: FT/IR-4600 type A instrument in either solid state or oil phase, with wavenumbers reported in cm^{-1}.

^{1}H and ^{13}C NMR spectra: Varian Mercury 300 MHz spectrometer (Oxford, UK) operating at 300.07 MHz and 75.4614 MHz for ^{1}H and ^{13}C, respectively. Chemical shifts are reported in ppm relative to Me_4Si used as the internal standard, and coupling constants (*J* values) in Hz. Typical conditions for ^{1}H spectra include a pulse width of 32°, acquisition time of 5 seconds, FT size of 32 K, digital resolution of 0.3 Hz per point, and the number of scans ranging from 1200 to 10,000 per spectrum. For ^{13}C spectra, typical conditions include a pulse width of 60°, FT size of 60 K, digital resolution of 0.6 Hz per point, and the number of scans varied accordingly.

ESI-MS: Waters/Micromass (Manchester, UK) ZQ mass spectrometer equipped with a Harvard Apparatus (Saint Laurent, Canada) syringe pump, with mass-to-charge ratio (m/z) reported. Sample solutions were prepared in MeOH at a concentration of approximately 10^{-5} M. Standard ESI-MS mass spectra were recorded at a cone voltage of 90 V.

2. Copies of NMR spectra

5

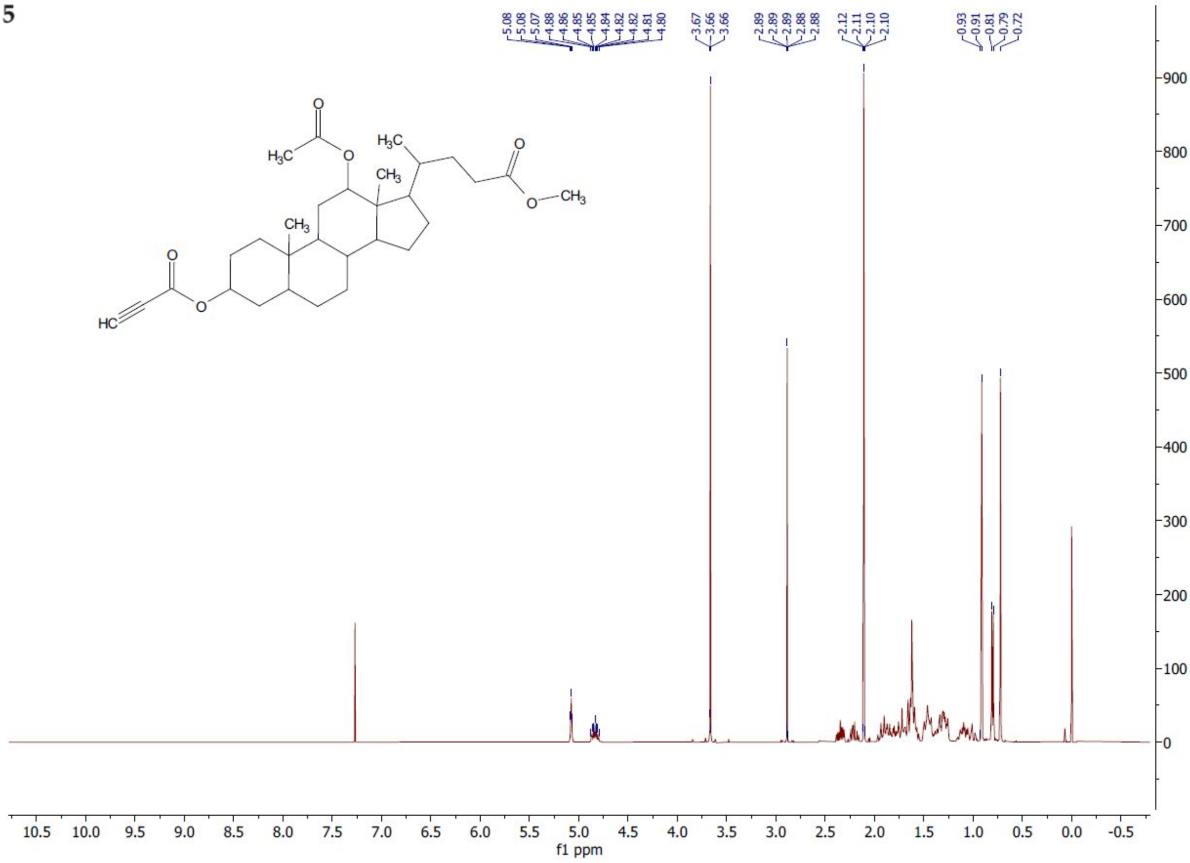

5

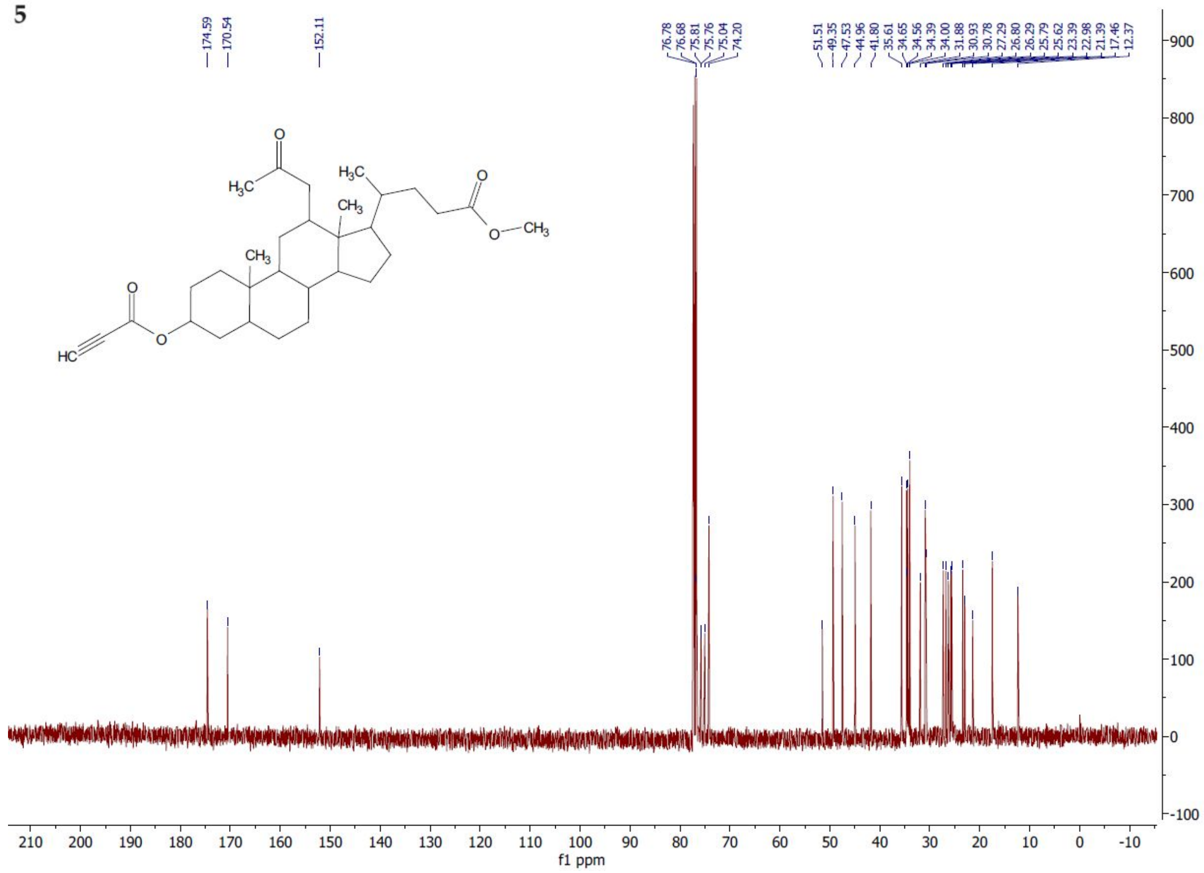

6

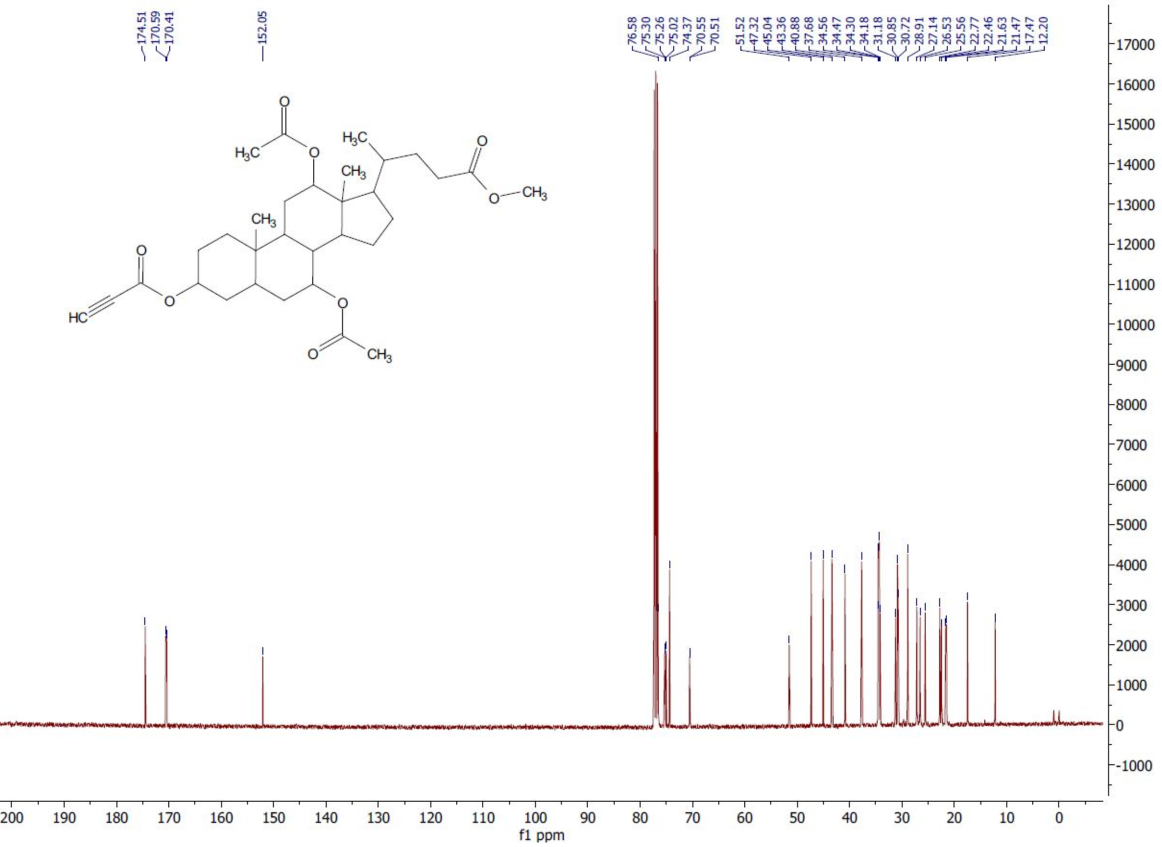
174.51
170.59
170.41
152.05
76.58
75.30
75.26
75.02
74.37
70.55
70.51
51.52
47.32
45.04
43.36
40.88
37.68
34.56
34.47
34.30
34.18
31.18
30.85
30.72
28.91
27.14
26.53
25.56
22.77
22.46
21.63
21.47
17.47
12.20
f1 ppm

6

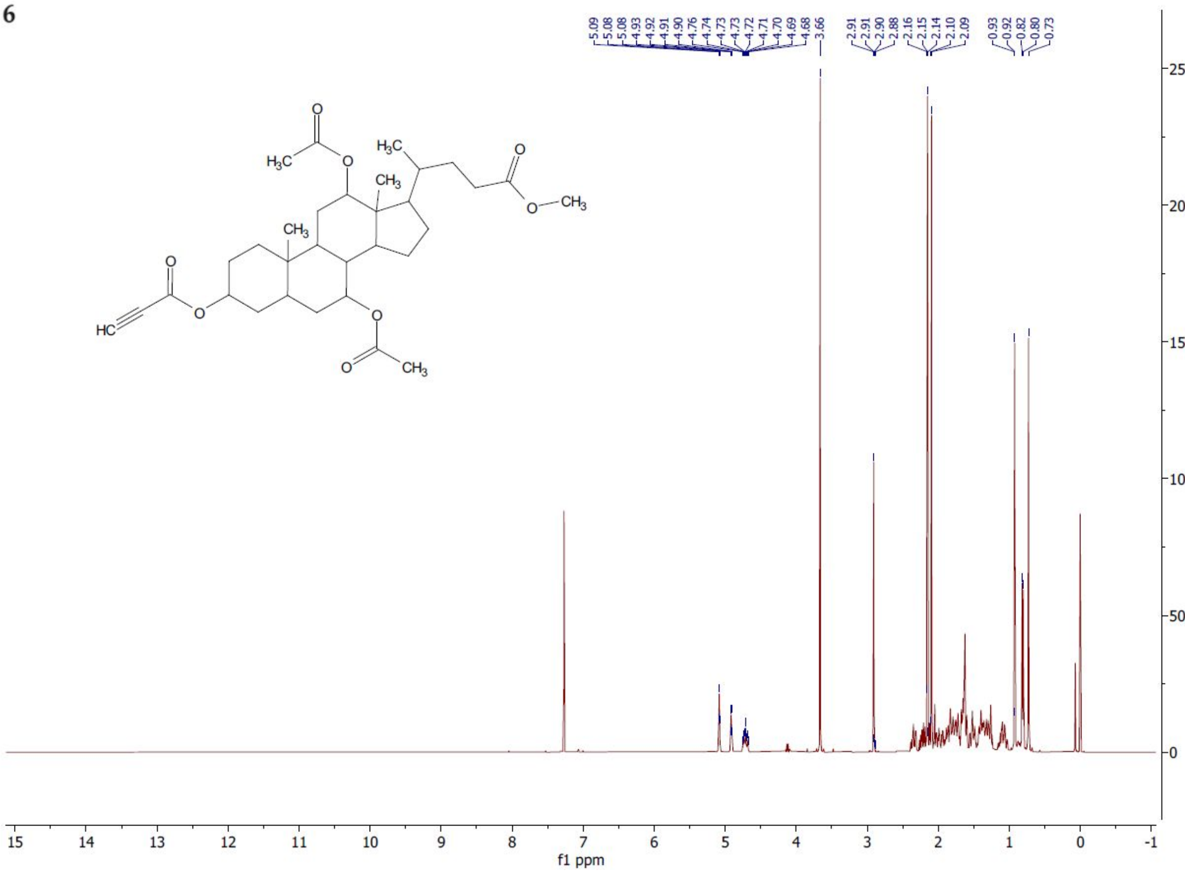
5.09
5.08
5.08
4.93
4.92
4.91
4.90
4.76
4.74
4.73
4.73
4.72
4.71
4.70
4.69
4.68
3.66
2.91
2.91
2.90
2.88
2.16
2.15
2.14
2.10
2.09
0.93
0.92
0.82
0.80
0.73
f1 ppm

9

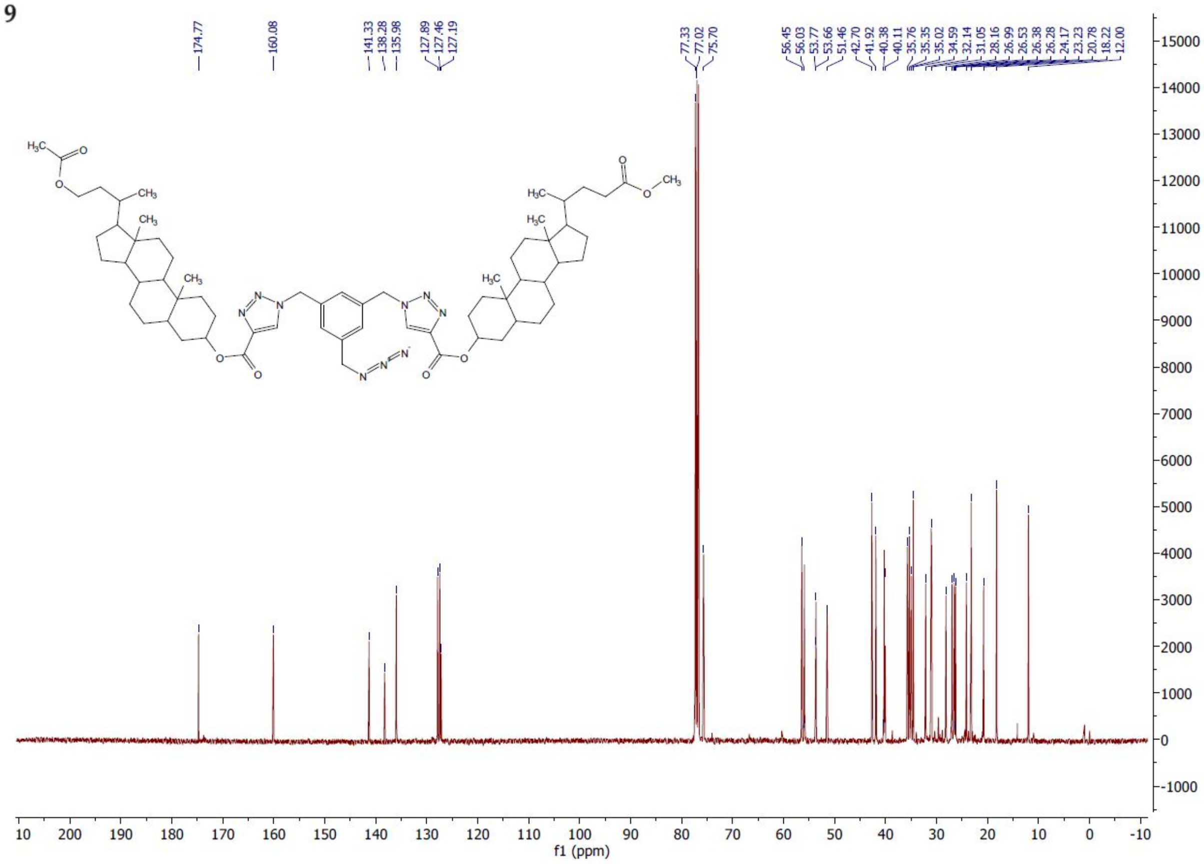

174.77
160.08
141.33
138.28
135.98
127.89
127.46
127.19
77.33
77.02
75.70
56.45
56.03
53.77
53.66
51.46
42.70
41.92
40.38
40.11
35.76
35.35
35.02
34.59
32.14
31.05
28.16
26.99
26.53
26.38
26.28
24.17
23.23
20.78
18.22
12.00
f1 (ppm)

9

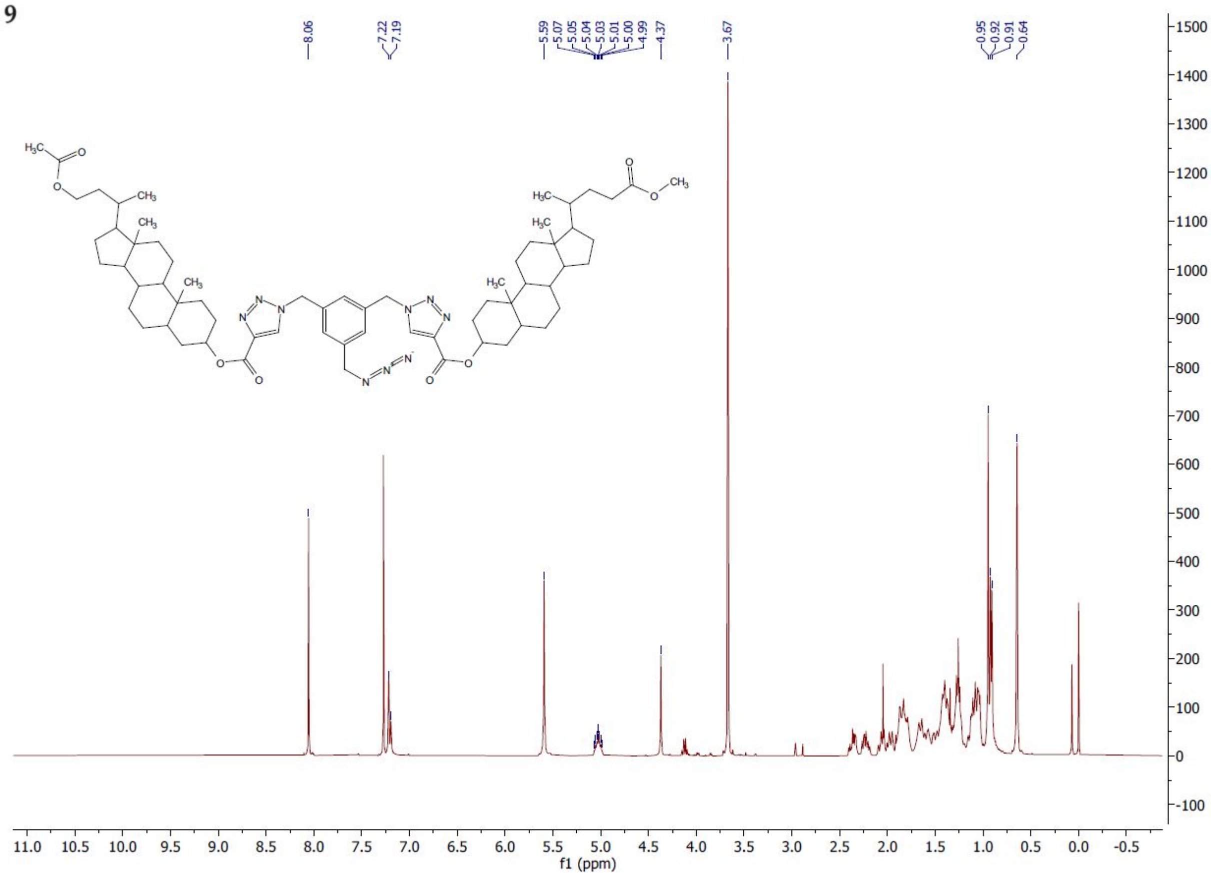

8.06
7.22
7.19
5.59
5.07
5.05
5.04
5.03
5.01
5.00
4.99
4.37
3.67
0.95
0.92
0.91
0.64
f1 (ppm)

10

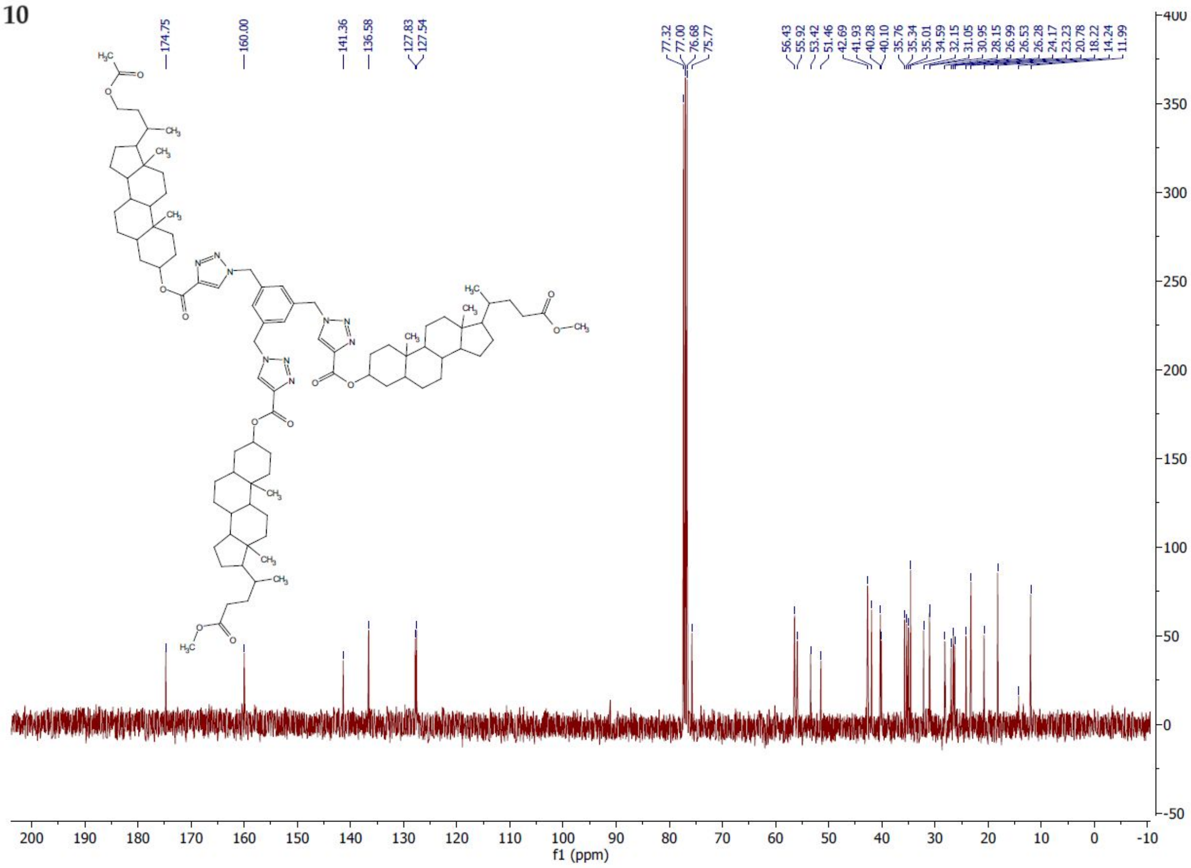

10

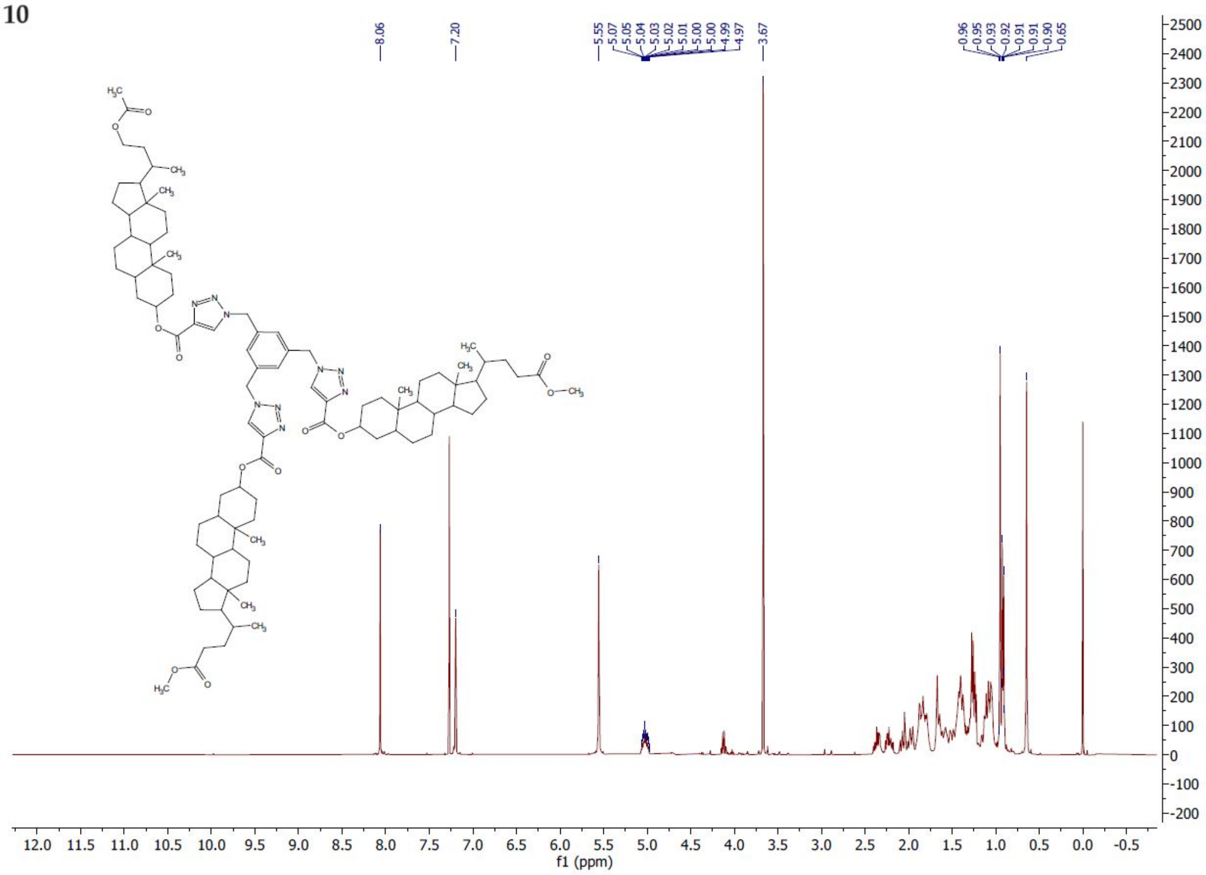

11

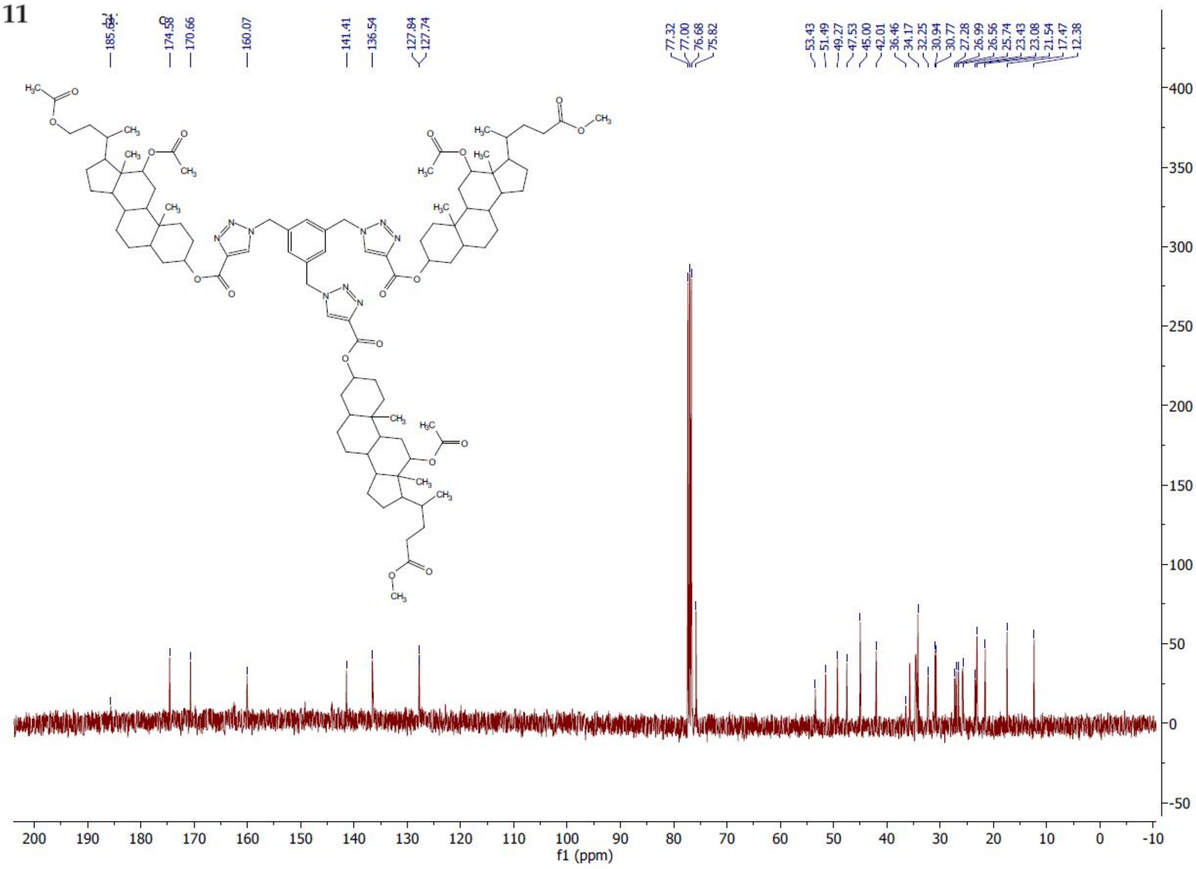
174.58
170.66
160.07
141.41
136.54
127.84
127.74
77.32
77.00
76.68
75.82
53.43
51.49
49.27
47.53
45.00
42.01
36.46
34.17
32.25
30.94
30.77
27.28
26.99
26.56
25.74
23.43
23.08
21.54
17.47
12.38
f1 (ppm)

11

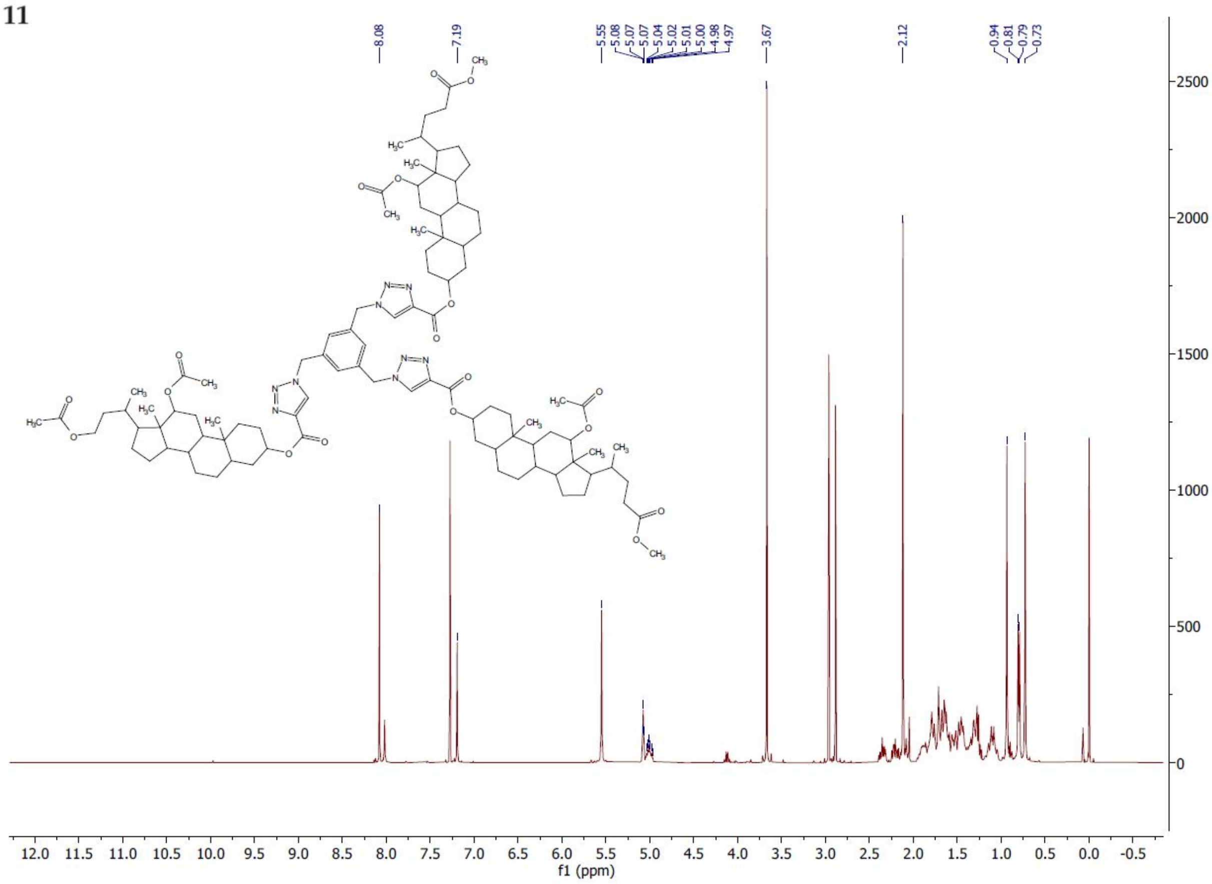
8.08
7.19
5.55
5.08
5.07
5.04
5.02
5.01
5.00
4.98
4.97
3.67
2.12
0.94
0.81
0.79
0.73
f1 (ppm)

12

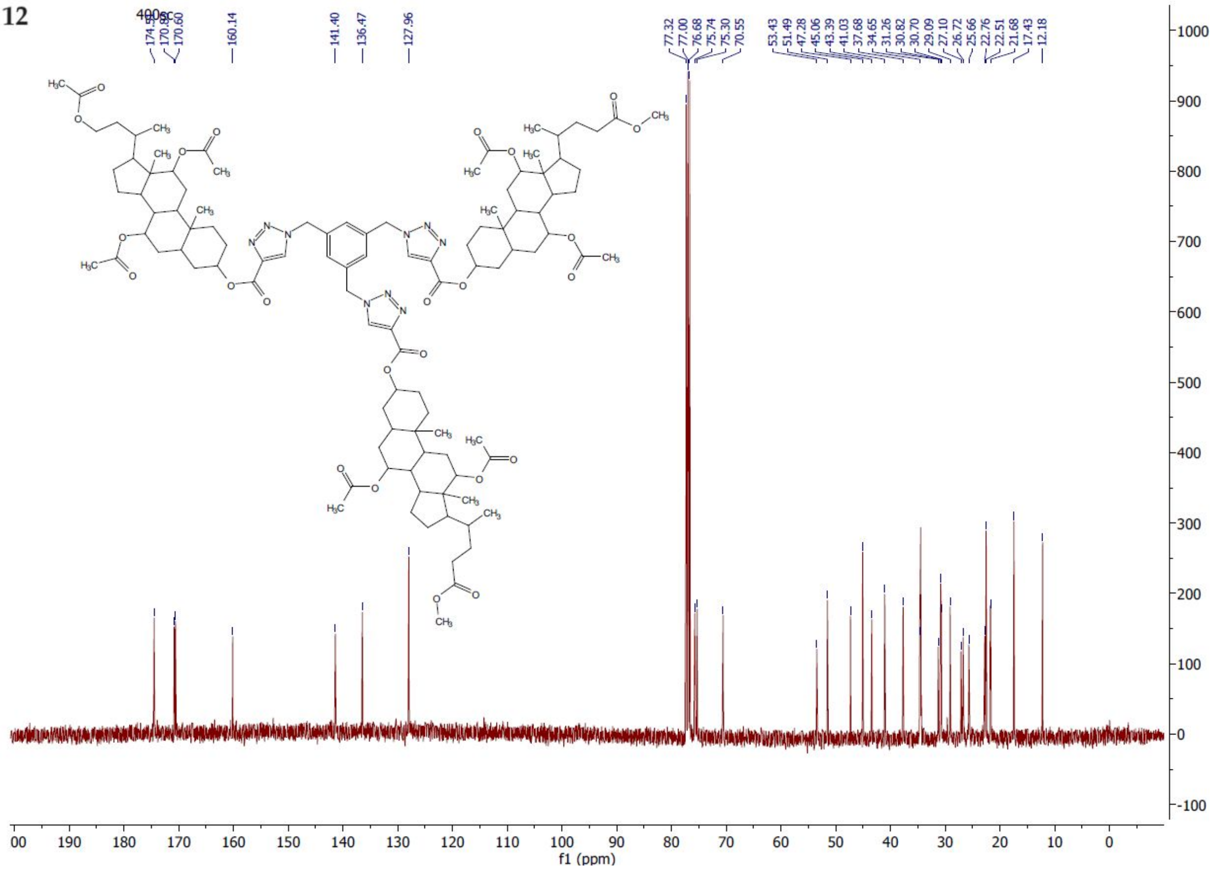

12

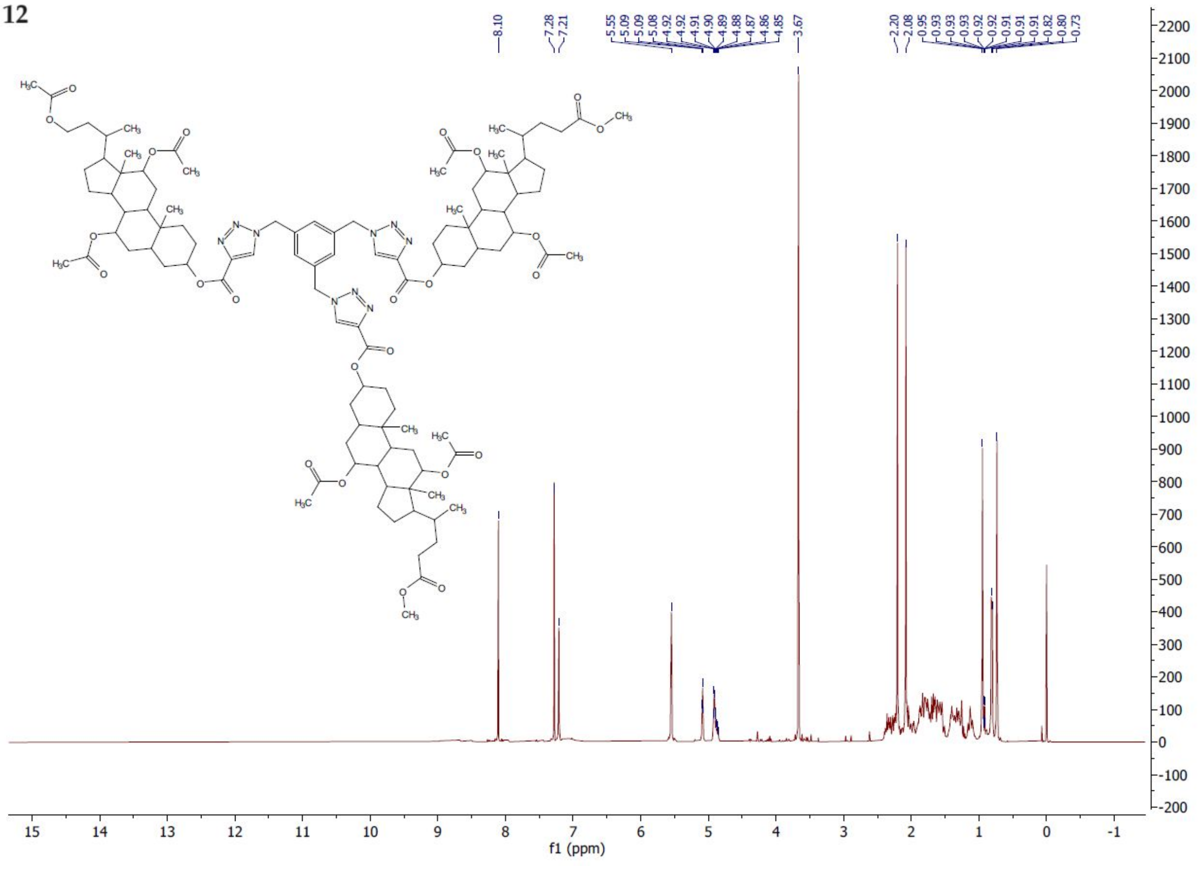

S-Figures 1. ^{1}H NMR and ^{13}C NMR spectra of compounds (**5**, **6**, **9–12**).

3. Copies of ESI-MS spectra

5

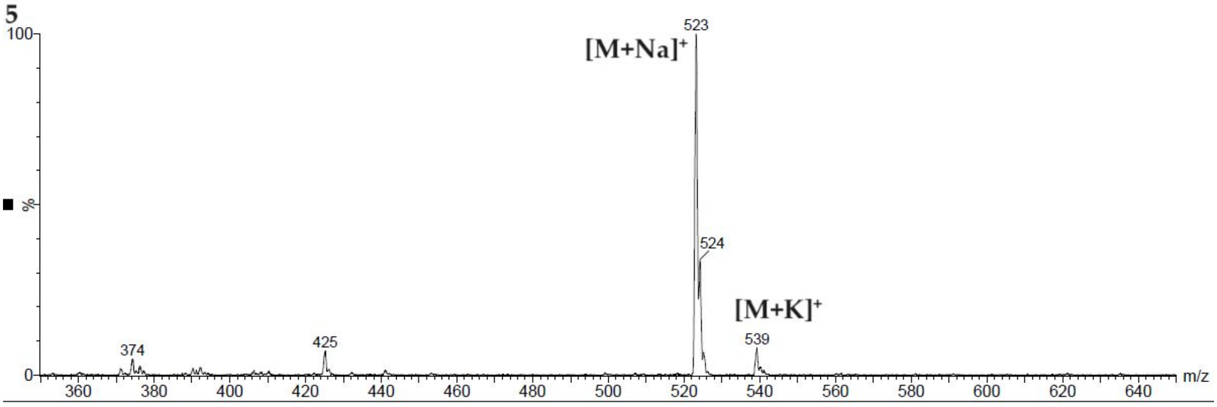

6

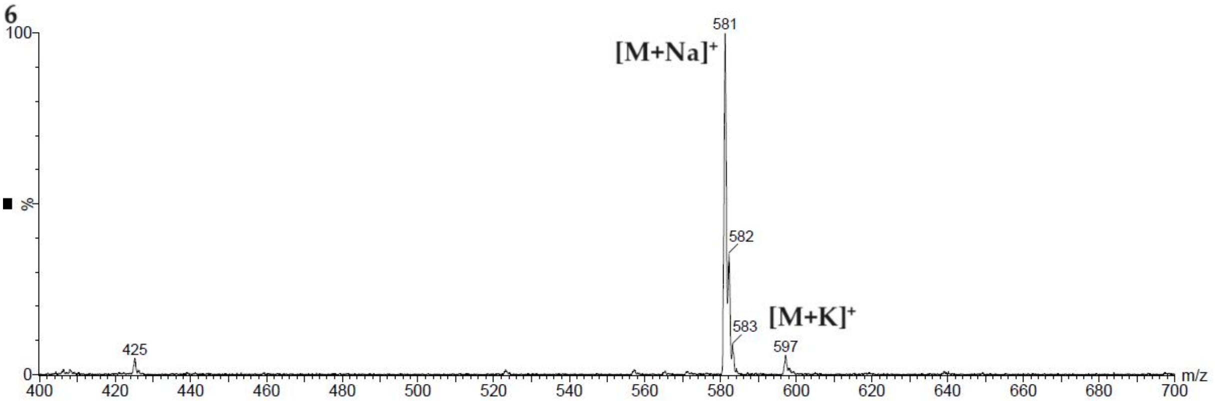

9

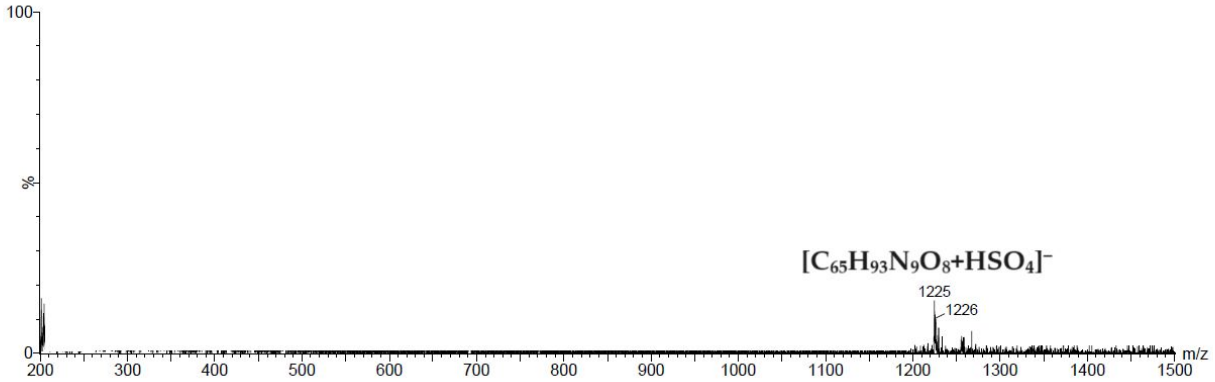

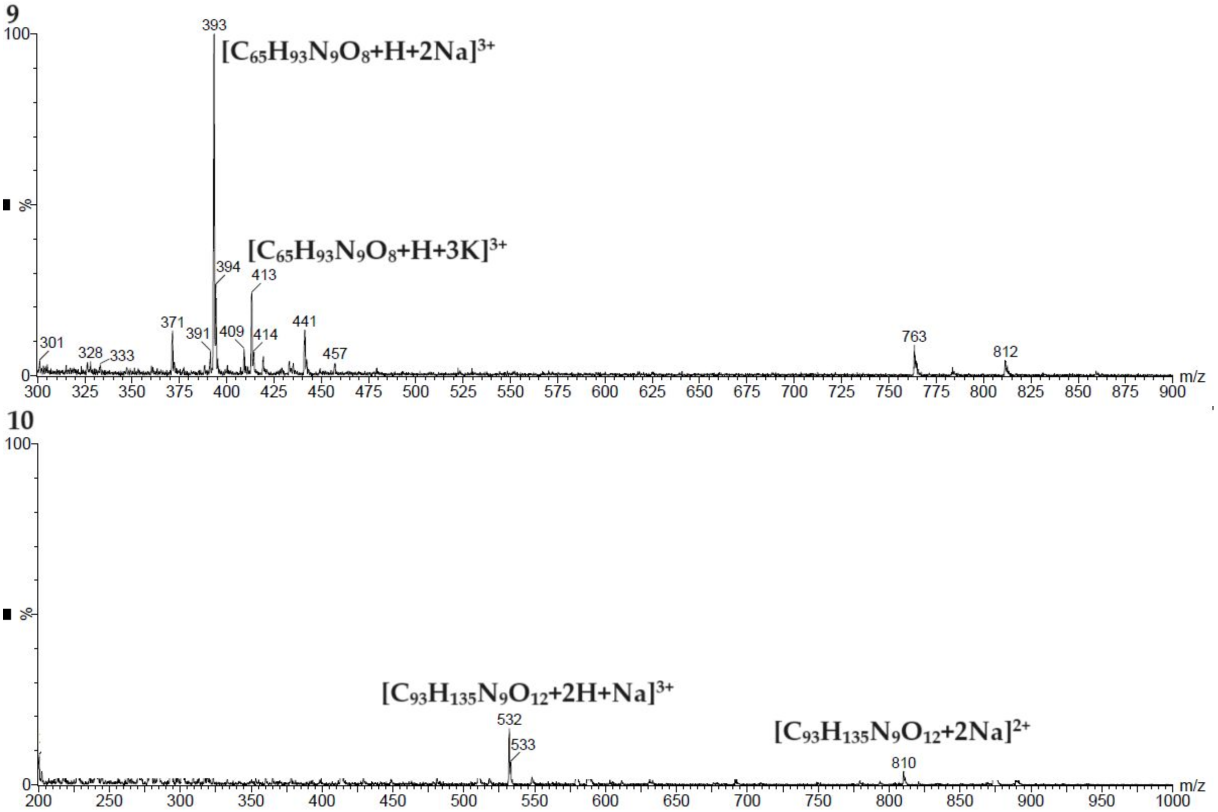

S-Figure 2. ESI-MS spectra compounds (**5**, **6**, **9**, **10**).

4. Copies of FT-IR spectra

5

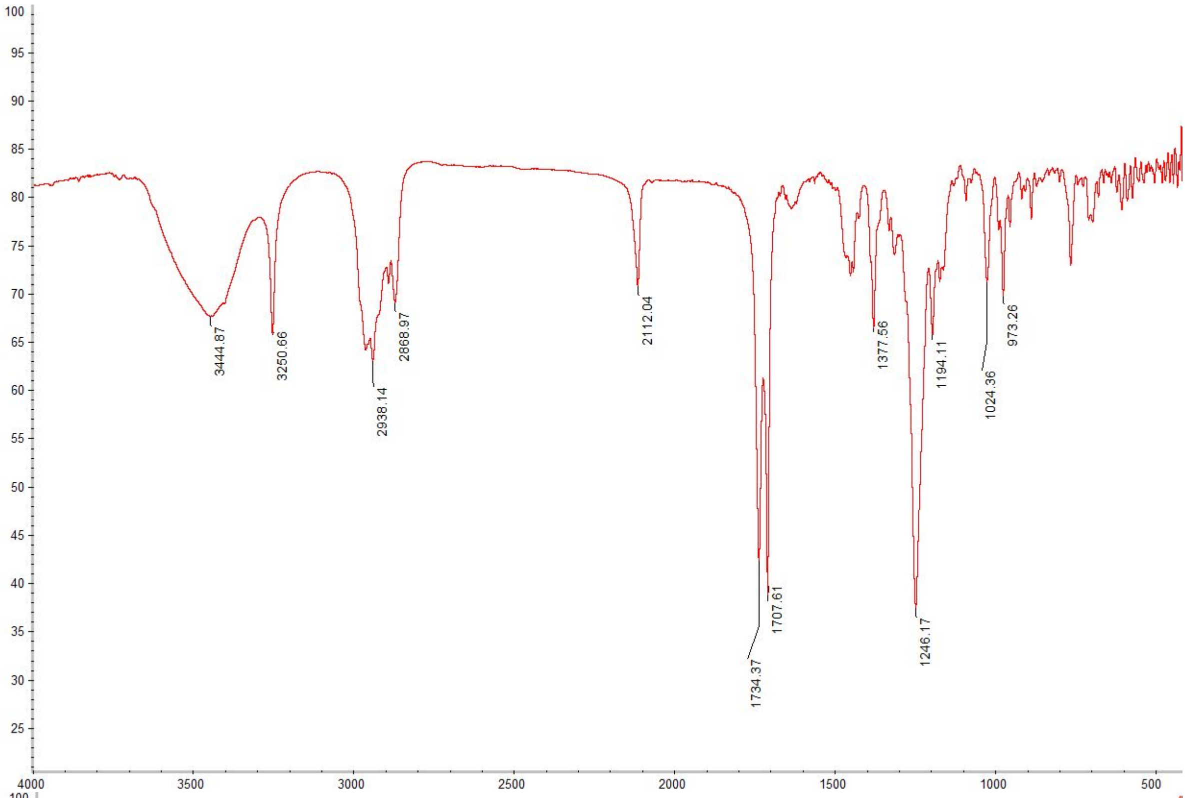

6

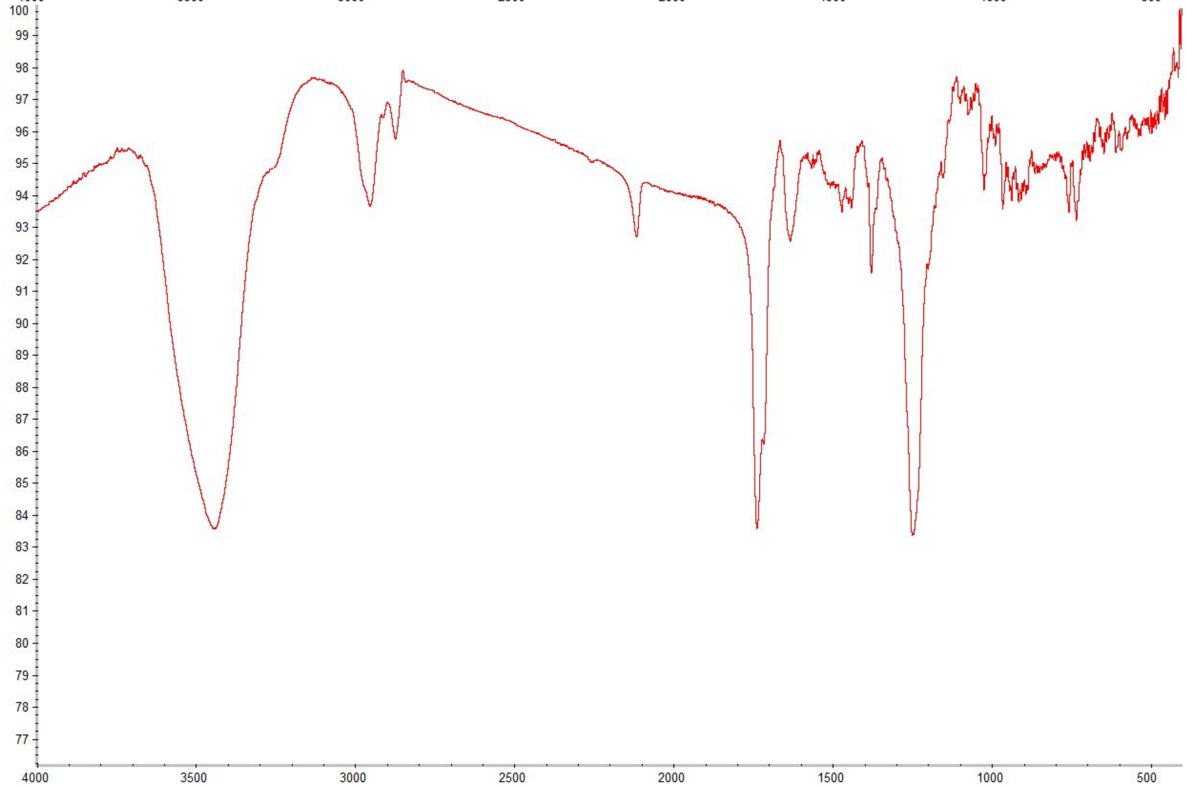

10

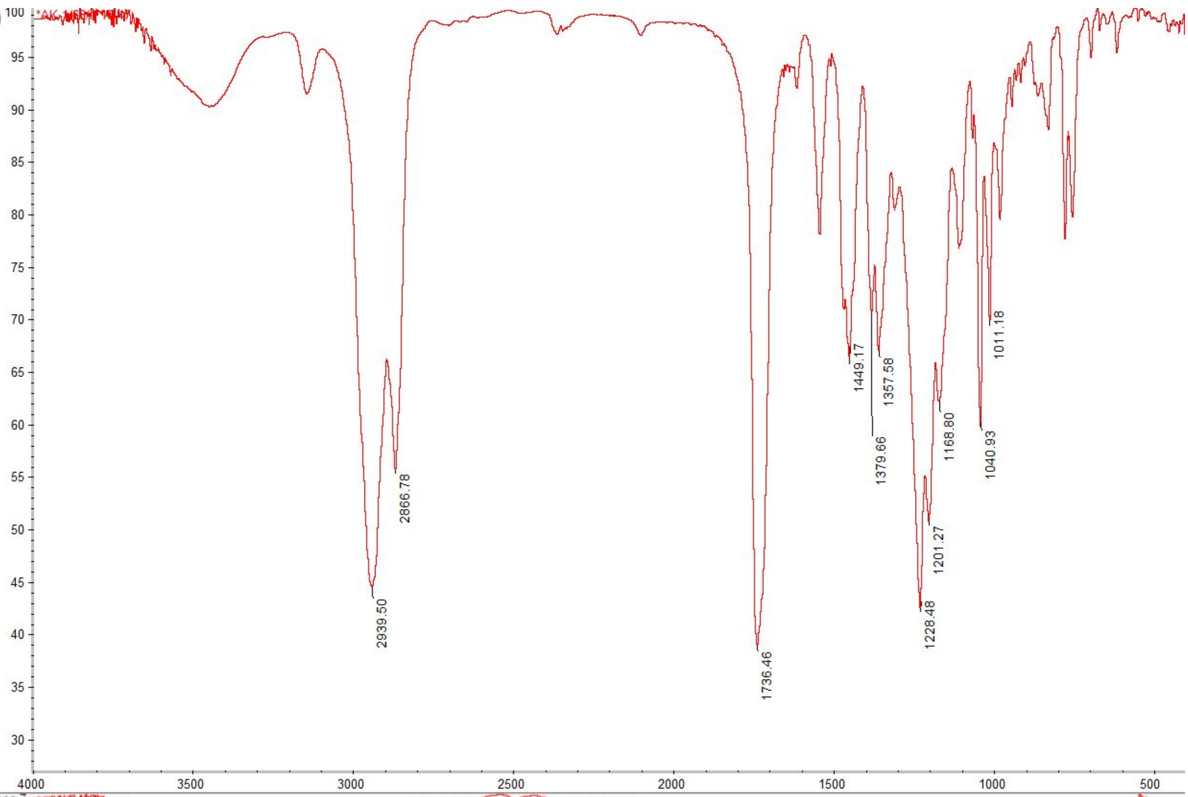
2939.50
2866.78
1736.46
1449.17
1379.66
1357.58
1228.48
1201.27
1168.80
1040.93
1011.18
100
95
90
85
80
75
70
65
60
55
50
45
40
35
30
4000
3500
3000
2500
2000
1500
1000
500

11

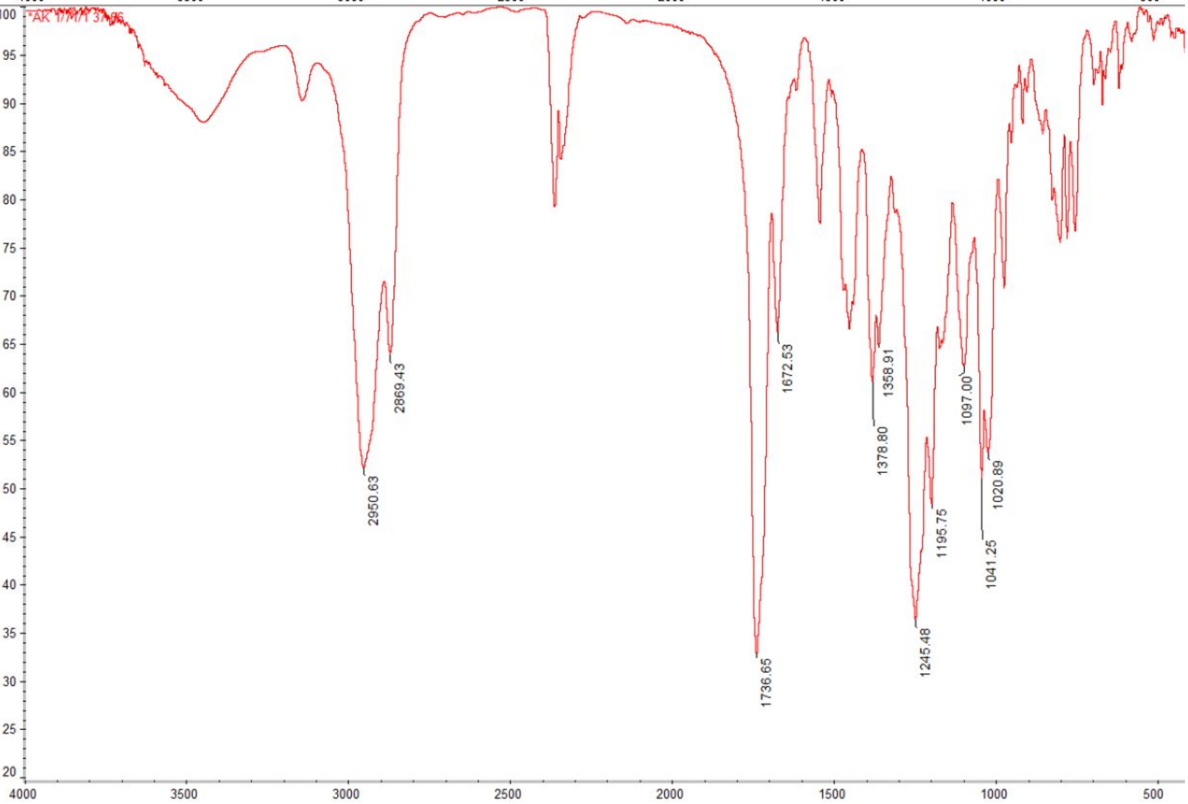
2950.63
2869.43
1736.65
1672.53
1378.80
1358.91
1245.48
1195.75
1097.00
1041.25
1020.89
100
95
90
85
80
75
70
65
60
55
50
45
40
35
30
25
20
4000
3500
3000
2500
2000
1500
1000
500

12

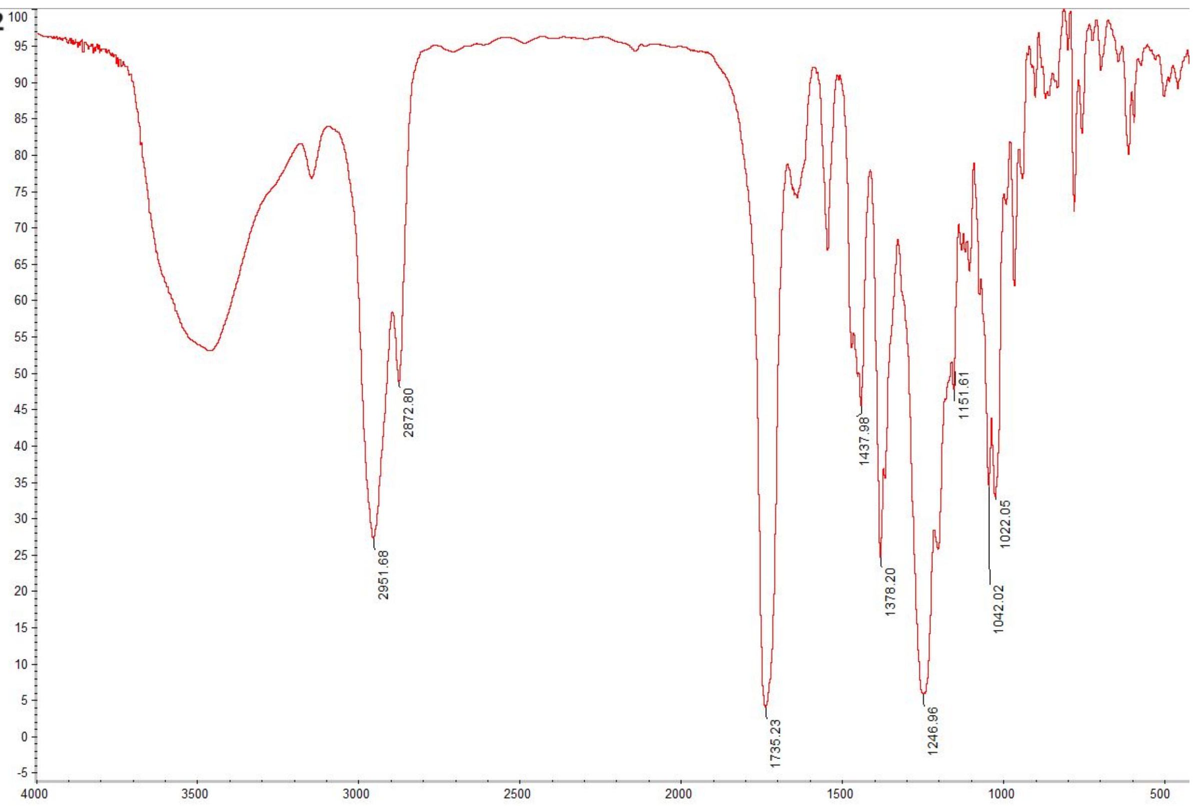

S-Figure 3. FT-IR spectra compounds (**5**, **6**, **9**, **10**, **11**, **12**).
